# Kidney Outcomes in Offspring Exposed to Hypertensive Disorders of Pregnancy: A Systematic Review and Meta-Analysis

**DOI:** 10.3390/jcm15207816

**Published:** 2026-10-10

**Authors:** Limei Xiong, Yong Zhang, Yuanxi Liao, Yao Zhang, Yanxia Mao, Tao Xiong

**Affiliations:** 1 Department of Paediatrics, West China Second University Hospital, Sichuan University, Chengdu 610041, China; lmxiong@scu.edu.cn (L.X.); zhangyaoaoo@163.com (Y.Z.); myxash@163.com (Y.M.); 2 Key Laboratory of Birth Defects and Related Diseases of Women and Children, Ministry of Education, Sichuan University, Chengdu 610041, China; 3 Department of Neonatology, Sichuan Provincial Women’s and Children’s Hospital (Affiliated Women’s and Children’s Hospital of Chengdu Medical College), Chengdu 610045, China; drzhang2021@126.com; 4 Huize County Maternal and Child Health Hospital, Qujing 654200, China; 18187441110@163.com; 5 Children’s Medicine Key Laboratory of Sichuan Province, Chengdu 610041, China

**Keywords:** hypertensive disorders of pregnancy, offspring, acute kidney injury, congenital anomalies of the kidney and urinary tract, chronic kidney disease

## Abstract

**Background:** Hypertensive disorders of pregnancy (HDP) may impair placental function and disrupt foetal nephrogenesis, increasing kidney vulnerability in offspring. However, the magnitude and phenotype-specific pattern of offspring kidney risk remain uncertain. We aimed to systematically assess the association between maternal HDP and offspring kidney outcomes. **Methods:** PubMed, EMBASE, Cochrane Library, Wanfang Data, and China National Knowledge Infrastructure were searched from inception to October 2025. Cohort and case–control studies evaluating maternal HDP and offspring kidney outcomes were included. Outcomes were grouped into three final categories, including chronic kidney disease (CKD), acute kidney injury (AKI), and congenital anomalies of the kidney and urinary tract (CAKUT). Two reviewers independently screened studies, extracted data, and assessed risk of bias. Pooled effect estimates were derived from random-effects meta-analysis using the generic inverse variance method, prioritising the adjusted estimates. The protocol was registered with PROSPERO (CRD420251175714). **Results:** Thirty studies (20 cohort and 10 case–control studies), including 10,954,573 participants, were included. Maternal preeclampsia was associated with an increased risk of offspring CKD (pooled adjusted relative effect 1.26, 95% CI 1.16, 1.37; I^2^ = 0%). Although crude analysis suggested a significant association between maternal HDP and offspring CAKUT (odds ratio [OR] 1.18, 95% CI 1.07, 1.31; I^2^ = 18%), the pooled adjusted estimate was not significant (adjusted OR 1.07, 95% CI 0.87, 1.31; I^2^ = 0%). A pooled adjusted estimate for offspring AKI was not derived, as this was reported in only 2 studies with considerable population heterogeneity. The overall crude risk ratio (RR) showed no statistically significant association but substantial heterogeneity (pooled crude RR 0.82, 95% CI 0.61, 1.10; I^2^ = 78%). Crude AKI RRs varied by criteria: Kidney Disease: Improving Global Outcomes 0.71 (95% CI 0.55, 0.91; I^2^ = 51%) versus Acute Kidney Injury Network 1.09 (95% CI 0.60, 1.96; I^2^ = 83%). **Conclusions:** Maternal HDP, particularly preeclampsia, was associated with elevated risks of offspring CKD; however, the pooled adjusted estimate for CAKUT was not statistically significant. Evidence for offspring AKI remains inconclusive, providing no reliable evidence for either an increased or decreased risk. Prospective studies using harmonised definitions and robust confounding control are needed to assess these associations and determine their clinical relevance.

## 1. Introduction

Hypertensive disorders of pregnancy (HDP) comprise a group of pregnancy-related hypertensive disorders including preeclampsia–eclampsia, gestational hypertension, chronic hypertension (pre-existing), and chronic hypertension with superimposed preeclampsia. These disorders are associated with adverse short- and long-term outcomes for both mother and offspring [1], affecting approximately 8% to 10% of pregnancies worldwide. Preeclampsia (PE) remains a leading cause of maternal and perinatal morbidity and mortality [2,3]. Beyond its immediate obstetric consequences, HDP is increasingly recognised as a condition with implications extending across the life course.

From the maternal perspective, HDP involves multisystem end-organ dysfunction, with the kidney representing a major target organ. Acute manifestations include proteinuria, a reduced glomerular filtration rate that may progress to acute kidney injury (AKI), and thrombotic microangiopathy-like lesions associated with pregnancy [2,3,4]. Long-term studies have shown that women with a history of HDP, particularly PE, have an elevated risk of chronic kidney disease (CKD) and kidney failure [5]. These findings are biologically coherent, as endothelial injury and microvascular dysfunction are central mechanisms underlying many kidney diseases, especially glomerular disorders, including those caused by thrombotic microangiopathy [6].

Mechanistically, HDP is increasingly conceptualised as placental and vascular disorders characterised by placental malperfusion and systemic endothelial injury [2,3]. Such alterations can impair uteroplacental blood flow and modify the intrauterine milieu through inflammation and oxidative stress. Within the framework of the Developmental Origins of Health and Disease hypothesis [7], disturbances in the intrauterine environment may have lasting effects on organ development. The kidney is particularly vulnerable because nephrogenesis continues into late gestation; disruption during this critical window may reduce nephron endowment and increase susceptibility to kidney disease later in life [3,8].

Adverse perinatal outcomes frequently associated with HDP may represent key pathways linking maternal disease to offspring renal vulnerability. Preterm birth, foetal growth restriction, and low birthweight occur more commonly in pregnancies complicated by HDP and are independently associated with reduced nephron number and increased long-term risk of CKD [8,9]. These factors may function as mediators, confounders, or both, complicating interpretation of observational data and underscoring the importance of careful analytic adjustment.

Against this biological background, several epidemiological studies have examined associations between maternal HDP and offspring kidney outcomes, including AKI, CKD, and congenital anomalies of the kidney and urinary tract (CAKUT) [10,11,12,13]. However, findings across studies remain inconsistent, partly owing to heterogeneity in exposure definitions, outcome ascertainment, follow-up duration, and control of shared familial and perinatal factors. Consequently, the magnitude, specificity, and independence of these associations remain uncertain.

To address these uncertainties and provide a comprehensive synthesis of the available evidence, we conducted a systematic review and meta-analysis to evaluate whether maternal HDP is associated with increased risk of offspring kidney outcomes. Because of high clinical heterogeneity of kidney diseases overall, we focused on three specific outcome categories: AKI, CKD, and CAKUT.

## 2. Materials and Methods

We conducted and reported this systematic review and meta-analysis in accordance with the Preferred Reporting Items for Systematic Reviews and Meta-Analyses reporting guidelines. The protocol was prospectively registered in the International Prospective Register of Systematic Reviews (PROSPERO registration identifier: CRD420251175714). As Supplementary Material you can find the PRISMA checklist. The original protocol prespecified AKI, CKD, CAKUT, and any kidney disease as outcomes. During data extraction, we found that the broad category of “any kidney disease” had substantial clinical and definitional heterogeneity. It was therefore removed from the quantitative and narrative syntheses on 4 June 2026, at which stage no quantitative meta-analysis had yet been performed. A composite glomerular and proteinuric outcome was added at the same time as a post hoc exploratory outcome. In response to peer-review concerns regarding its overlap with CKD, this outcome was removed on 7 August 2026, after completion of the primary analyses for AKI, CKD, and CAKUT. This removal did not affect the eligibility criteria, data extraction, or effect estimates for the three outcomes. The final review therefore focused exclusively on AKI, CKD, and CAKUT. All modifications were methodology-driven rather than results-driven, and their exact timing and rationales are documented in the updated PROSPERO record and Supplementary Table S1. This study is based on previously published data and publicly available materials. As it involved no direct interaction with human subjects or collection of primary identifiable data, ethical approval and informed consent were not required.

### 2.1. Data Sources and Search Strategy

A systematic search of five electronic databases, including PubMed, EMBASE, Cochrane Library, Wanfang Data and China National Knowledge Infrastructure, was undertaken from inception to 31 October 2025. Medical Subject Headings and relevant free-text terms were used for HDP, kidney disease, and CAKUT. The detailed PubMed search strategy is provided in the Supplementary Table S2. Corresponding key Chinese terms were used in Chinese databases. We selected publications without restrictions of origin, country or language. Reference lists of relevant reviews and included original studies were also screened for additional eligible studies.

### 2.2. The Inclusion and Exclusion Criteria

The inclusion criteria were as follows: (1) case–control studies or cohort studies (prospective or retrospective); (2) published an original effect estimate or provided sufficient data to calculate the association between any form of HDP (specifically PE, gestational hypertension, or chronic hypertension in pregnancy) and at least one of the kidney outcomes in offspring.

The exclusion criteria were as follows: (1) non-human studies; (2) case reports, case series, letters, commentaries, notes and editorials; (3) studies that used changes in biological or laboratory markers of kidney function (e.g., change in estimated glomerular filtration rate or microalbuminuria) as their sole outcome measure, without providing information about the diagnosis; (4) for AKI outcomes, studies that did not apply standardized diagnostic criteria (e.g., Kidney Disease: Improving Global Outcomes [KDIGO] or Acute Kidney Injury Network [AKIN] criteria).

Potentially overlapping cohorts were systematically identified by comparing study settings, recruitment or birth years, cohort names, registry data sources, eligibility criteria, sample sizes, participating institutions, authorship, follow-up durations, and baseline characteristics. Publications with potentially overlapping populations were not treated as independent studies. For each underlying cohort, we retained one primary publication according to the largest eligible sample size, the longest or most complete follow-up, the most comprehensive outcome ascertainment, and the availability of directly relevant adjusted estimates. Each cohort contributed only one prespecified kidney outcome and was not reused across CKD, AKI, or CAKUT analyses. Therefore, no cohort was counted more than once in the quantitative synthesis.

### 2.3. Definition and Classification of HDP Exposures

Under contemporary international guidelines, HDP includes preeclampsia/eclampsia, gestational hypertension, chronic (pre-existing) hypertension, and chronic hypertension with superimposed preeclampsia. Because the included studies used heterogeneous and historical terminology, exposures were classified according to the definitions reported in the original studies rather than retrospectively reclassified according to current criteria. Pregnancy-induced hypertension was considered equivalent to gestational hypertension only when the original definition clearly described pregnancy-onset hypertension and excluded preeclampsia/eclampsia. When subtypes could not be distinguished, exposures were retained as overall HDP.

### 2.4. Outcomes Assessment

Offspring kidney outcomes were defined according to each study’s diagnostic criteria, including registry-based ICD codes, hospital records, or established clinical definitions. We focused on three primary outcome categories, defined as follows:

1. CKD, defined by persistently reduced estimated glomerular filtration rate (<60 mL/min/1.73 m^2^) and/or markers of kidney damage (e.g., albuminuria) lasting ≥3 months, or equivalent source-defined ICD-coded diagnoses. An individual with estimated glomerular filtration rate ≥ 90 mL/min/1.73 m^2^ was classified as CKD G1 only when a qualifying marker of kidney damage, such as persistent albuminuria, was documented for at least 3 months.
2. AKI, defined by study-specific criteria, preferentially the KDIGO or AKIN definitions.
3. CAKUT, defined by structural congenital anomalies by imaging after birth, or equivalent ICD-coded diagnoses.

Outcome categories were assigned according to the primary endpoint definition used in each original study, rather than by reclassifying patients based on underlying etiologies. Accordingly, glomerulonephritis was not treated as a separate outcome. For studies that reported both CKD and glomerular disease, we extracted only the source-defined CKD effect estimate.

The primary analyses focused on adjusted effect estimates (odds ratio [OR], risk ratio [RR], or hazard ratio [HR]) comparing offspring of mothers with HDP with offspring of mothers without HDP. Secondary analyses examined crude (unadjusted) effect estimates for each specific outcome category.

### 2.5. Causal Framework and Covariate Selection

The primary objective of this meta-analysis was to estimate the total effect of maternal HDP on offspring kidney outcomes, encompassing both direct pathways and indirect pathways potentially mediated through perinatal factors. A conceptual directed acyclic graph was constructed to formalize the assumed causal structure (Figure S1).

In accordance with this conceptual causal framework, maternal characteristics were considered potential baseline confounders and were prioritized in the primary adjustment set. Conversely, perinatal factors, including gestational age, birthweight, fetal growth restriction, and neonatal complications, were classified as potential intermediate variables (mediators) lying downstream of maternal HDP. Because adjustment for intermediate variables may block part of the mediated pathway or induce collider bias under some causal structures, we prioritised models controlling for maternal characteristics and other baseline confounders without conditioning on these potential perinatal mediators [14]. When a study reported only a single adjusted model that already included perinatal covariates, that estimate was retained in the primary analysis to avoid discarding data. However, these estimates were interpreted as conditional associations rather than unbiased total-effect estimates. Thus, the pooled adjusted estimates should be interpreted as estimates intended to approximate the total effect, while acknowledging that the adjustment strategies varied across studies. In addition, because very-low-birth-weight infants or very preterm infants differ significantly from other neonates in terms of renal development, perinatal complications, and baseline risk, studies based on such populations were not included in the primary analysis to avoid the interference of clinical heterogeneity on the pooled estimates. Subgroup analyses by gestational age, birthweight, and adjustment strategy were treated as exploratory investigations of heterogeneity rather than causal mediation or direct-effect analyses.

### 2.6. Data Extraction and Quality Assessment

Data from all eligible studies were extracted independently by two reviewers using a standardised data extraction form. Extracted items included the author, publication year, population source, study design, follow-up duration, exposure and outcome definition, sample size, confounders adjusted for (if any) and crude and adjusted measures of association.

Author contact was required in only one study, where a markedly asymmetric confidence interval was queried but no response was received [15]. Where effect estimates were unavailable, absolute numbers of events were extracted and crude measures of association were calculated. Discrepancies were resolved by discussion with a senior investigator.

Included studies were independently appraised using the Newcastle–Ottawa Scale (NOS) for assessing the quality of non-randomized studies in meta-analyses [16]. Two reviewers performed the quality assessment, with disagreements resolved by consensus. Study quality was considered in interpreting the findings, but studies were not excluded solely based on NOS scores. Studies scoring 7–9 points were categorized as having relatively high methodological quality. To complement the NOS assessment, we additionally used the Risk Of Bias In Non-randomized Studies of Exposures (ROBINS-E) tool to assess domain-specific risk of bias [17]. Assessments were conducted separately for studies evaluating CKD, CAKUT, and AKI. Two reviewers independently assessed bias due to confounding, measurement of the exposure, selection of participants, post-exposure interventions, missing data, measurement of the outcome, and selection of the reported result. Disagreements were resolved through discussion and consensus. ROBINS-E domain-level judgments were used to identify methodological limitations that might not be captured by the NOS summary score, while the NOS remained the primary quality assessment framework for consistency with previous systematic reviews.

### 2.7. Statistical Analysis and Sensitivity Analysis

For each of the three outcome categories (AKI, CKD, and CAKUT), we performed separate random-effects meta-analyses to pool associations between maternal HDP and offspring kidney outcomes. Crude and adjusted effect estimates were synthesised using the generic inverse-variance method on the log scale, and results were presented as pooled estimates with 95% confidence intervals (CIs). Statistical heterogeneity was assessed using the I^2^ statistic. Following Cochrane Collaboration guidance, we considered I^2^ values of 0–40% as possibly unimportant. Heterogeneity was interpreted by I^2^ and clinical heterogeneity across studies. In the adjusted primary analyses, potential sources of heterogeneity were explored through subgroup analyses stratified by adjustment model (maternal confounder-only versus potential perinatal mediators such as gestational age, birth weight, or neonatal complications), offspring age (0–18 years versus >18 years), and HDP subtype. For crude (unadjusted) analyses, we conducted prespecified meta-regression and subgroup analyses by HDP subtype, offspring age, gestational age, and birth weight. Tests for subgroup differences were performed to compare effect estimates across strata. Sensitivity analyses were performed using leave-one-out analyses and restriction to high methodological quality studies. Potential publication bias and small-study effects were evaluated using funnel plots and Begg’s and Egger’s tests when sufficient studies were available.

For AKI, because the outcome is not rare in certain cohorts (e.g., neonatal intensive care), the primary meta-analysis pooled RRs. OR was synthesised separately as a sensitivity analysis. For CKD, where event rates are low, the rare disease assumption applies. Under this assumption, ORs were considered approximately equivalent to RRs [18,19,20]. HRs incorporate time-to-event information and therefore represent a different estimand from cumulative-risk RRs. Because the included CKD studies reported different effect measures, the reported ORs and HRs were synthesized in the primary analysis as a summary relative association, rather than as an exact RR. Where possible, analyses restricted to studies reporting the same effect measure were conducted as sensitivity analyses. For CAKUT, all included studies reported ORs, which were pooled directly.

All analyses were conducted using Review Manager (RevMan, Version 5.2, The Nordic Cochrane Centre, Copenhagen, Denmark) and Stata 12.0 (StataCorp, College Station, TX, USA). RevMan was used to conduct the primary meta-analyses and generate the corresponding forest plots, whereas Stata was used to perform the meta-regression analyses, Egger’s and Begg’s tests. R (version 4.5.0; R Foundation for Statistical Computing, Vienna, Austria) with the metafor package was utilized to reproduce the identical DerSimonian–Laird random-effects models and output high-resolution vector forest plots for the sensitivity analyses. The certainty of evidence was assessed using the Grading of Recommendations Assessment, Development and Evaluation (GRADE) approach [21], and was graded as high, moderate, low, or very low.

## 3. Results

### 3.1. Study Selection

A total of 4080 records were identified through database searches, and 21 additional reports were identified through citation searching. After the removal of 464 duplicate records, 3616 records underwent title and abstract screening, of which 3485 were excluded. Among the 131 database-derived reports assessed for full text, 105 were excluded for the following reasons: overlapping or duplicate cohort publications reporting the same eligible outcomes (n = 6), no diagnosis of offspring AKI, CKD, or CAKUT after birth (n = 83), nephrotic syndrome without the required 3-month chronicity for CKD (n = 1) [22], no eligible comparison group (n = 11), and AKI not defined according to KDIGO or AKIN criteria (n = 4). Among the 21 reports identified through citation searching, 17 were excluded because they did not evaluate maternal hypertensive disorders as the exposure of interest (n = 15) or did not provide original data (n = 2). Finally, 30 studies were included in the systematic review [9,10,11,12,15,23,24,25,26,27,28,29,30,31,32,33,34,35,36,37,38,39,40,41,42,43,44,45,46,47]. The process of study selection is outlined in Figure S2.

This systematic review included a total of 20 cohort studies and 10 case–control studies, which were conducted in different regions worldwide, including the United States, Europe, China, Japan and other places. The characteristics of each study are summarised in Table 1. Sample size ranged from 50 to 4,186,615 participants. Follow-up across included studies ranged from several days after birth to 50 years. However, for the outcome of offspring AKI, all studies were restricted to neonatal populations. According to the NOS assessment, the methodological quality varied by study design and outcome. Studies evaluating CKD and CAKUT generally received NOS scores of 7–9 and were categorized as having relatively high methodological quality. However, the majority of cohort studies on AKI received a score of 6, primarily due to insufficient representativeness of exposed cohorts and lack of adjustment for key confounders (Supplementary Tables S3 and S4). Consistent with the finding, the supplementary ROBINS-E assessment classified all 18 AKI studies as having high overall risk of bias, primarily due to inadequate adjustment for important potential confounders; 16 studies also raised concerns regarding participant selection because they mainly included preterm and/or low-birth-weight infants. The detailed ROBINS-E assessment for studies evaluating CKD, CAKUT, and AKI are presented separately in Figures S3–S5.

### 3.2. Primary Outcomes: Adjusted Associations Between Maternal HDP and Offspring Kidney Outcomes

#### 3.2.1. Offspring CKD

**Figure 1:**
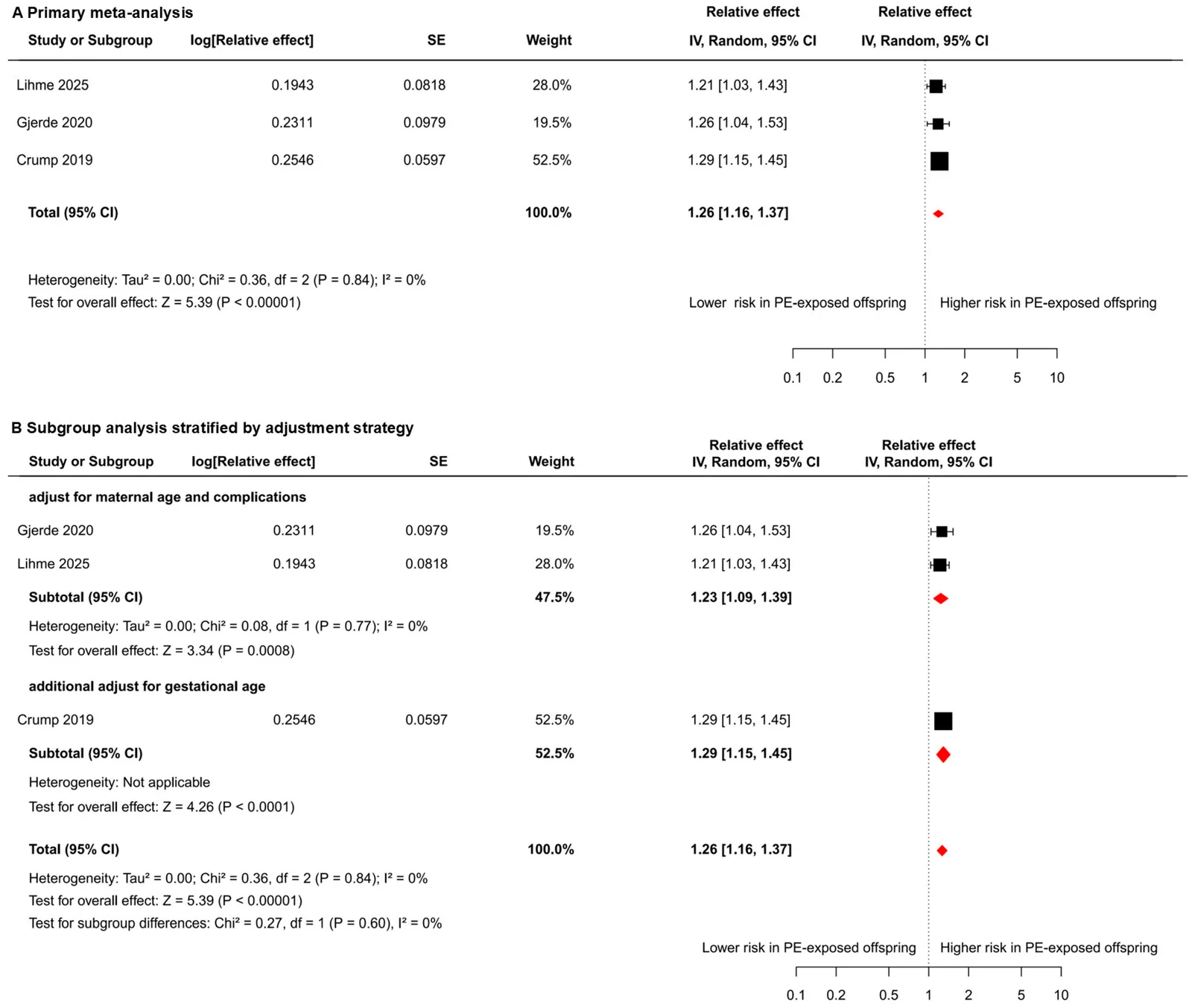
Forest plot of adjusted associations between preeclampsia and offspring chronic kidney disease [9,10,25]. Marker sizes reflect the weight assigned to each study (inverse variance method), and horizontal lines represent 95% confidence intervals. Because the included chronic kidney disease studies reported different effect measures, the reported odds ratios and hazard ratios were synthesized in the primary analysis as a summary relative association. CI, confidence interval; IV, inverse variance; PE, preeclampsia; SE, standard error.

Four studies reported adjusted estimates for offspring CKD following maternal PE, with offspring ages ranging from 0 to 50 years [9,10,15,25]. But one study was based on very-low-birth-weight infants and reported an adjusted estimate of 1.05 (95% CI 1.01, 1.66) [15]. This study was retained and analysed separately to avoid introducing clinical heterogeneity into the primary analysis. Therefore, three nationwide cohort studies were included in the meta-analysis [9,10,25], with a total of 9,138,214 participants across the original cohorts. The pooled adjusted relative effect was 1.26 (95% CI 1.16, 1.37; I^2^ = 0%) (Figure 1A). Sensitivity analyses (leave-one-out) confirmed robustness (Figure S6A).

To assess the influence of the effect measure, we conducted sensitivity analyses restricted to studies reporting the same measure of association. The HR-only analysis (2 studies) yielded a pooled adjusted HR of 1.26 (95% CI 1.15, 1.39), whereas only one study reported an adjusted OR of 1.26 (95% CI 1.04, 1.53). These estimates were consistent in direction and broadly similar in magnitude to those of the primary analysis.

When stratified by adjustment strategy, two studies adjusted for maternal age and diseases; the pooled adjusted relative effect was 1.23 (95% CI 1.09, 1.39; I^2^ = 0%). The third study, additionally adjusted for gestational age, reported the adjusted relative effect of 1.29 (95% CI 1.15, 1.45) (Figure 1B) [9].

Only two studies reported adjusted effect estimates for CKD outcomes within the childhood age window. One reported the adjusted HR (aHR) in a general population sample for offspring aged <1 year (aHR 0.93; 95%CI 0.48, 1.80) [10] and the other reported the adjusted OR (aOR) in children aged < 10 years with a history of very-low-birth-weight (aOR 1.05; 95% CI 1.01, 1.66) [15]. Given the distinct populations, no pooled childhood-specific estimate was calculated. One study also provided an estimate restricted to adulthood (aged >24 years; aHR 1.36; 95%CI 1.06,1.74) [10].

#### 3.2.2. Offspring CAKUT

**Figure 2:**
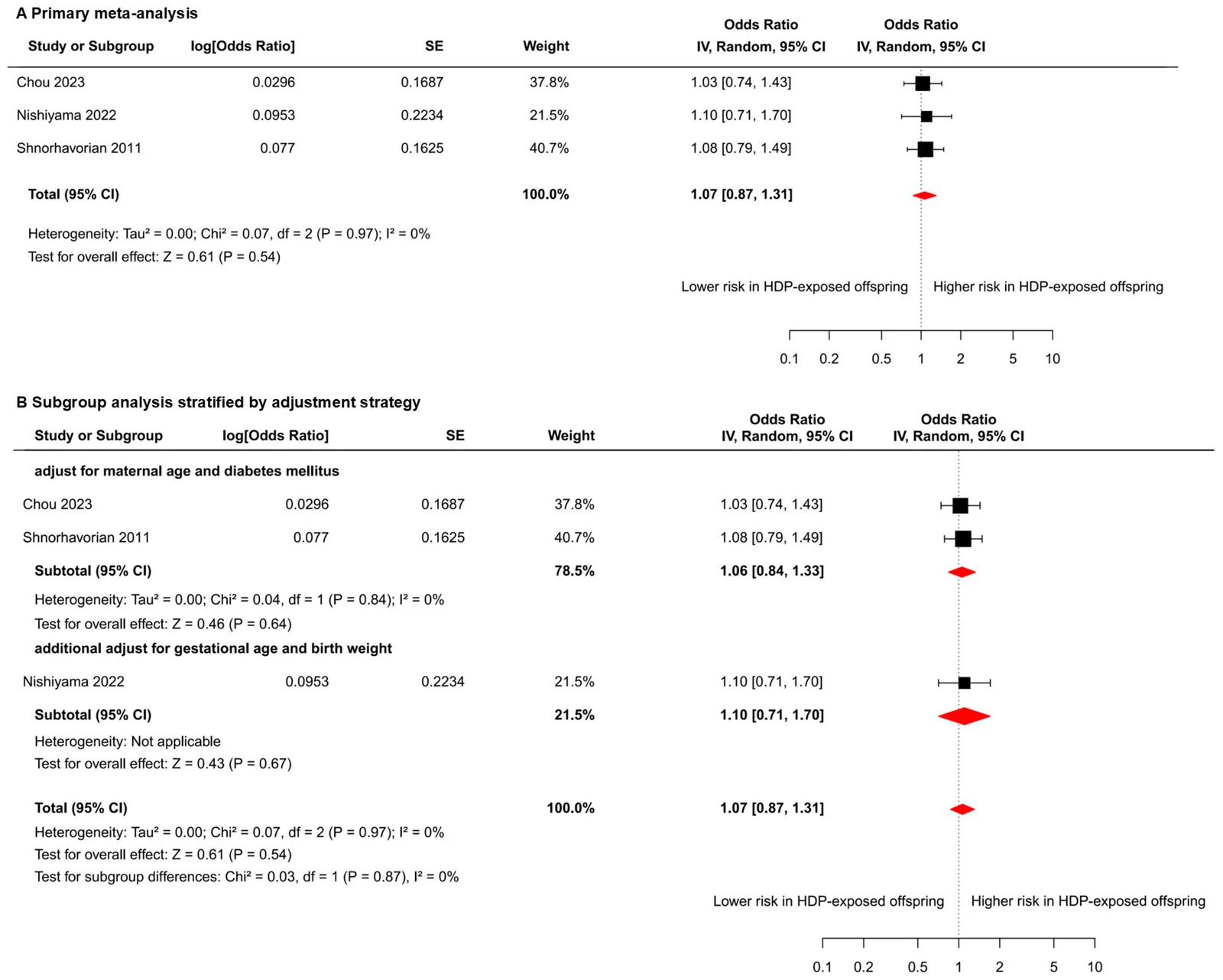
Forest plot of adjusted associations between hypertensive disorders of pregnancy and offspring congenital anomalies of the kidney and urinary tract [11,28,47]. Marker sizes reflect the weight assigned to each study (inverse variance method), and horizontal lines represent 95% confidence intervals. CI, confidence interval; HDP, hypertensive disorders of pregnancy; IV, inverse variance; SE, standard error.

Although four studies reported the adjusted effect estimates for offspring CAKUT in mothers with HDP versus those without [11,28,29,47], one study presented separate aORs for different CAKUT subtypes and could not be included in the primary pooled estimate [29]. Therefore, 3 studies enrolling 1,752,310 participants, with offspring aged from birth to 5 years, were included in the meta-analysis. The pooled aOR was 1.07 (95% CI 0.87, 1.31; I^2^ = 0%) (Figure 2A). The sensitivity analysis confirmed similar results (Figure S6B).

When stratified by adjustment strategy, two studies adjusted for maternal age and gestational diabetes mellitus; the pooled aOR was 1.06 (95% CI 0.84, 1.33; I^2^ = 0%). The third study, additionally adjusted for gestational age and birth weight, reported the aOR of 1.10 (95% CI 0.71, 1.70) (Figure 2B) [28].

When stratified by HDP subtypes, two studies defined maternal exposure broadly as hypertension without distinguishing pre-pregnancy chronic hypertension from pregnancy-induced hypertension. The third study was restricted to women with chronic hypertension (present before pregnancy). Therefore, formal mutually exclusive subgroup analysis by HDP subtypes was not feasible. The pooled aOR of two studies was 1.05 (95% CI 0.81, 1.37; I^2^ = 0%) [11,28]. The third study reported the aOR of 1.08 (95% CI 0.79, 1.49) [47].

#### 3.2.3. Offspring AKI

Two studies reported adjusted effect estimates for AKI in offspring of mothers with HDP versus those without [12,40]. However, one study was based on extremely low-birth-weight preterm infants [40], which reported an aOR of 0.37 (95% CI 0.14, 0.97). The other study included infants of all gestational ages and reported an aOR of 0.87 (95% CI 0.63, 1.21) [12]. Because of substantial clinical heterogeneity between the two study populations (e.g., differences in gestational age and baseline risk), we did not perform a meta-analysis for this outcome.

### 3.3. Secondary Outcomes: Crude Associations Between Maternal HDP and Offspring Kidney Outcomes

#### 3.3.1. Offspring CKD

**Figure 3:**
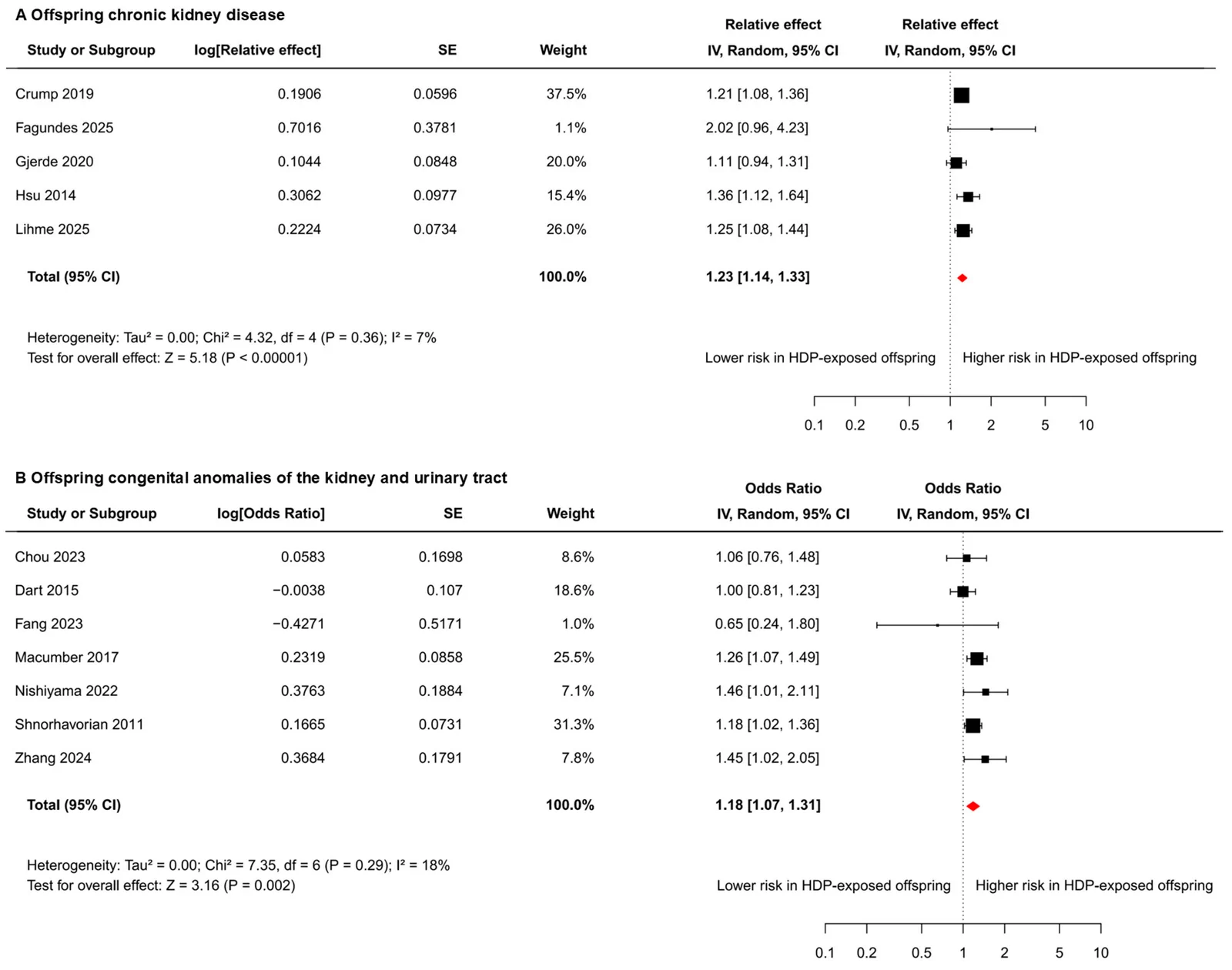
Forest plot of crude associations between hypertensive disorders of pregnancy and two offspring kidney outcomes [9,10,11,15,23,24,25,26,27,28,29,47]. Marker sizes reflect the weight assigned to each study (inverse variance method), and horizontal lines represent 95% confidence intervals. Because the included chronic kidney disease studies reported different effect measures, the reported odds ratios and hazard ratios were synthesized in the primary analysis as a summary relative association. CI, confidence interval; HDP, hypertensive disorders of pregnancy; IV, inverse variance; SE, standard error.

Five studies including 9,158,868 participants reported crude associations between maternal HDP and offspring CKD [9,10,15,25,26]. The pooled crude relative effect was 1.23 (95% CI 1.14, 1.33; I^2^ = 7%) (Figure 3A). Excluding studies with high risk of bias, the pooled crude relative effect was 1.20 (95%CI 1.11, 1.30; I^2^ = 0%). The sensitivity analysis (leave-one-out) showed similar results (Figure S7A).

Sensitivity analyses restricted to studies reporting the same measure of association showed that the OR-only analysis (4 studies) yielded a pooled crude OR of 1.24 (95% CI 1.10, 1.40), whereas only one study reported a crude HR of 1.21 (95% CI 1.08, 1.36). These estimates were consistent in direction and broadly similar in magnitude to those of the primary analysis.

#### 3.3.2. Offspring CAKUT

Seven studies including 1,788,774 children reported crude associations between maternal HDP and offspring CAKUT [11,23,24,27,28,29,47]. The pooled crude OR was 1.18 (95% CI 1.07, 1.31; I^2^ = 18%) (Figure 3B). When restricted to 3 studies that reported both crude and adjusted estimates, the pooled crude OR was 1.19 (95% CI 1.05, 1.35; I^2^ = 0%). Excluding studies with high risk of bias, the pooled crude OR was 1.22 (95% CI 1.08, 1.37; I^2^ = 0%). The sensitivity analysis (leave-one-out) revealed similar results (Figure S7B).

#### 3.3.3. Offspring AKI

Eighteen studies reported the crude associations between exposure to maternal HDP and offspring AKI. Of these, 17 studies reported a crude RR, whereas the remaining one reported a crude OR (0.65, 95% CI 0.49, 0.87), which was examined separately [12]. The 17 studies, including 4769 children, were included in the meta-analysis. The pooled crude RR was 0.82 (95% CI 0.61, 1.10) with substantial heterogeneity (I^2^ = 78%) (Figure S8A). A sensitivity analysis showed that after removing low or moderate quality research (NOS scores < 7), the pooled crude RR was 0.75 (95% CI 0.54, 1.06) with low heterogeneity (I^2^ = 0%) (Figure S8B). Leave-one-out analysis showed that after removing the study by Bolat et al., 2013, the originally non-significant association became statistically significant with high heterogeneity (Figure S9) [33]. In the meta-regression analysis, we did not find any factors that accounted for heterogeneity in the pooled estimates (Supplementary Table S5).

**Figure 4:**
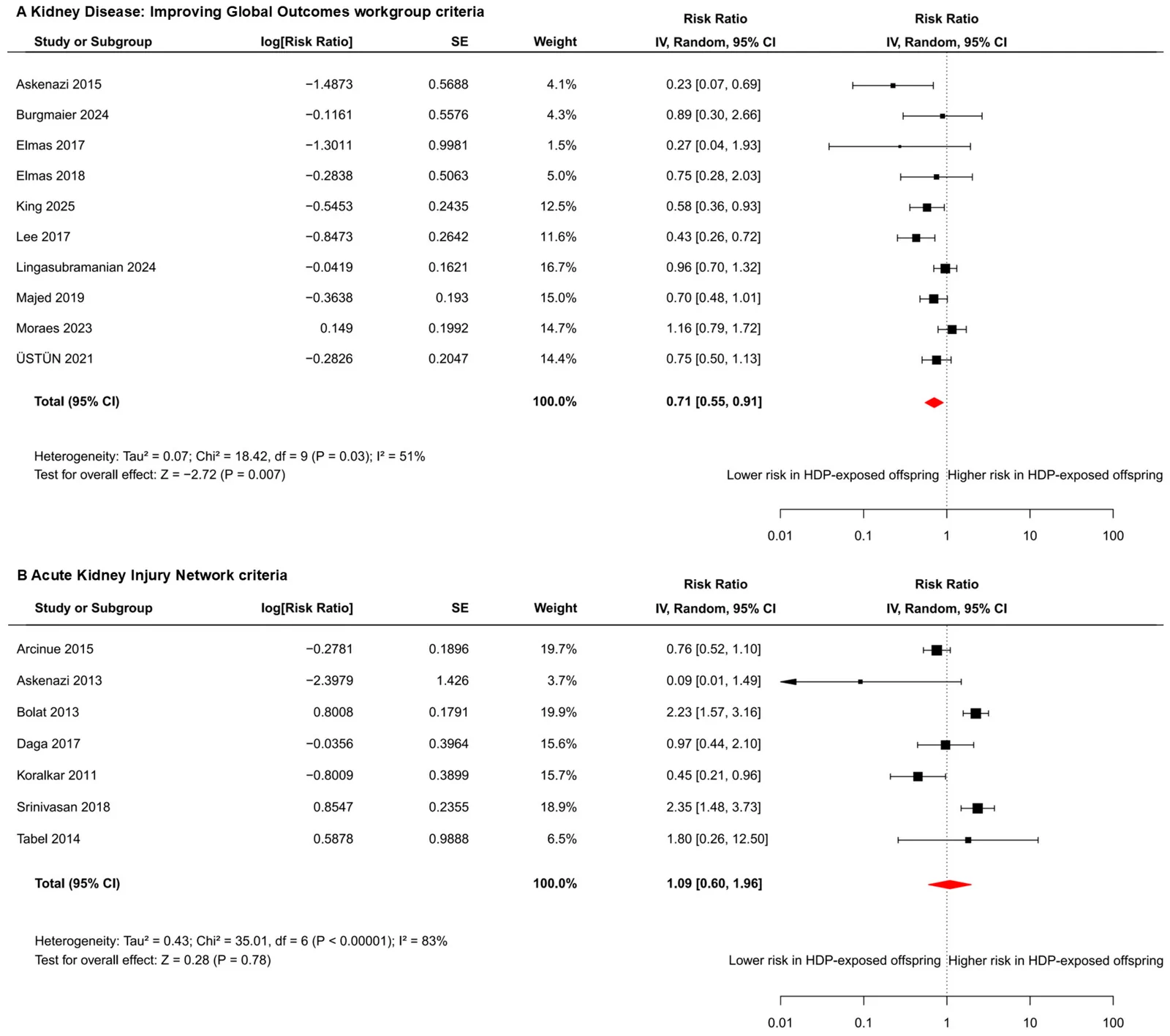
Forest plot of crude associations between hypertensive disorders of pregnancy and offspring acute kidney injury diagnosed according to two criteria [30,31,32,33,34,35,36,37,38,39,40,41,42,43,44,45,46]. Marker sizes reflect the weight assigned to each study (inverse variance method), and horizontal lines represent 95% confidence intervals. CI, confidence interval; HDP, hypertensive disorders of pregnancy; IV, inverse variance; SE, standard error.

AKI was diagnosed according to KDIGO or AKIN criteria. When stratified by diagnostic criteria, crude estimates differed descriptively between the two criteria. Ten studies used KDIGO criteria, and the pooled crude RR was 0.71 (95% CI 0.55, 0.91; I^2^ = 51%). Seven studies used AKIN criteria, and the pooled RR was 1.09 (95% CI 0.60, 1.96; I^2^ = 83%) (Figure 4). In a sensitivity analysis limited to studies with NOS ≥7, the pooled crude RR was 0.61 (95% CI 0.25, 1.48) for KDIGO and 0.78 (95% CI 0.54, 1.12) for AKIN, both with very low heterogeneity (I^2^ = 0%). Because all included AKI studies were assessed as having a high risk of bias, a sensitivity analysis excluding high-risk studies was not feasible.

#### 3.3.4. Exploratory Subgroup Analysis for Crude Estimates

Subgroup analyses of crude associations were performed according to hypertensive disorder subtype, offspring age (0–18 years versus >18 years), gestational age (<37 weeks versus ≥37 weeks), and birthweight (<1500 g versus ≥1500 g) (Table 2). These subgroup findings are exploratory and based on study-level data.

For CKD, only two studies reported crude estimates restricted to childhood [15,26], while the others were based on nationwide samples of all ages. Therefore, a formal subgroup analysis comparing age groups was not feasible. The pooled estimate for the two childhood studies alone was 1.40 (95% CI 1.14, 1.71; I^2^ = 2%). Because of limited studies, subgroup analysis for different birthweight was not available as well. Subgroup analysis for gestational ages showed that the association between PE and CKD was not significant among preterm populations (<37 weeks; OR 1.34, 95% CI 0.71, 2.51; I^2^ = 59%).

For CAKUT, only subgroup analysis by HDP subtypes was feasible. A significant association was observed between gestational hypertension and CAKUT (OR 1.23, 95% CI 1.11, 1.37; I^2^ = 0%). For AKI, because all studies reported outcomes restricted to childhood, subgroup analysis by offspring age could not be performed. In preterm infants (<37 weeks), an inverse association with AKI was found (RR 0.69, 95% CI 0.54, 0.88; I^2^ = 39%).

### 3.4. Publication Bias and GRADE Certainty of Evidence

Visual inspection of the funnel plot of crude effect estimates for AKI revealed no marked asymmetry (Supplementary Figure S10), and neither Egger’s test (*p* = 0.151) nor Begg’s test (*p* = 0.537) detected statistically significant funnel-plot asymmetry. However, given the modest number of included studies and the limited statistical power of these tests, publication bias or other small-study effects cannot be excluded. Because fewer than 10 included studies in the meta-analyses for other outcomes, particularly those based on adjusted estimates, funnel plots and statistical tests for funnel-plot asymmetry were not performed, and the potential impact of publication bias on these results also remains uncertain.

According to the GRADE framework, the certainty of evidence was rated as low for the adjusted association between maternal PE and offspring CKD, and very low for the adjusted association between maternal HDP and offspring CAKUT because of serious imprecision (Supplementary Table S6). All crude associations were rated as very low certainty mainly because of serious risk of bias, inconsistency, and/or imprecision.

## 4. Discussion

Previous systematic reviews focused on maternal renal outcomes after HDP or isolated offspring kidney structure [5,48]. To our knowledge, this is one of the most comprehensive meta-analyses to date examining the observational association between maternal HDP and offspring kidney outcomes, including CAKUT. The results suggest that maternal HDP—particularly PE—is associated with an increased risk of offspring CKD. In contrast, findings for AKI were inconsistent across diagnostic criteria and provided no reliable evidence of either an increased or decreased risk, deriving largely from unadjusted analyses. Moreover, after available confounder adjustment, the pooled adjusted CAKUT estimate was not statistically significant, and the certainty of this evidence was very low. Differences across outcomes and study settings suggest that the observed patterns reflect both perinatal biological pathways and methodological variation. Collectively, these findings suggest that maternal HDP is an observational marker associated with heightened offspring chronic kidney risk, though current evidence does not establish it as an independent causal determinant.

Our findings are relevant in the context of the growing global burden of CKD. CKD is now recognised as a major non-communicable disease contributing substantially to morbidity, premature mortality, and healthcare expenditure worldwide [49]. Identifying early-life risk markers that precede clinical diseases is therefore a public health priority. HDP is routinely documented in obstetric records and affects a sizeable proportion of pregnancies, making it a potentially scalable and low-cost marker for future epidemiological and health-services research.

Several biological mechanisms support the plausibility of this link. HDP is characterised by placental malperfusion and systemic endothelial dysfunction and is strongly linked to preterm birth, foetal growth restriction, and low birthweight [50,51]. Because nephrogenesis continues into late gestation, shortened gestation and intrauterine stress may reduce nephron endowment and limit functional reserve [52]. Compensatory hyperfiltration and nephron hypertrophy provide a framework for long-term CKD susceptibility. In addition, preterm infants are frequently exposed to hemodynamic instability and nephrotoxic medications in neonatal intensive care, which may further increase renal vulnerability and AKI risk [53]. Contemporary reviews also emphasise that kidney health consequences of HDP may extend across generations, with perinatal mechanisms likely contributing to this effect [3].

The most consistent finding across the included literature was the association between maternal PE and offspring CKD. Both adjusted and crude estimates were generally consistent across large population-based cohorts and remained similar in sensitivity analyses. However, the observed associations and their related exploratory subgroup analyses warrant cautious interpretation regarding perinatal factors such as gestational age. In our subgroup analyses, point estimates were broadly comparable between cohorts dominated by term births and those restricted to preterm births. Although the association reached statistical significance only in the term stratum, this difference in statistical significance does not establish effect modification. The formal test for subgroup differences was not statistically significant (*p* = 0.91), although its power was limited by the small number of studies. More fundamentally, gestational age and fetal growth are not merely background stratification variables; they are closely correlated, influenced by maternal HDP [54,55], and may act as intermediate mediators along the causal pathway to offspring kidney impairment. Because conventional observational analyses cannot readily disentangle direct maternal intrauterine effects from perinatal mediation without formal mediation frameworks, these subgroup findings must remain strictly exploratory. Our findings indicate that maternal PE is consistently associated with offspring CKD in observational cohorts, supporting its role as a potential early-life epidemiological risk marker. Future prospective studies with harmonized data and formal mediation modeling are needed to elucidate these causal pathways.

CAKUT represents one of the major causes of pediatric CKD. However, the present meta-analysis did not show a statistically significant association between maternal HDP and CAKUT after confounder adjustment. Thus, current evidence does not suggest that CAKUT is the primary pathway explaining the observed HDP–CKD association, although a modest contribution cannot be entirely ruled out given the very low certainty of the CAKUT evidence. Furthermore, in the subset of studies reporting both crude and adjusted estimates, the crude OR was attenuated after adjustment for confounders, suggesting that the apparent association may be partly explained by shared maternal metabolic or environmental factors, such as gestational diabetes mellitus [56]. Renal and urinary tract morphogenesis occurs during early gestation. Although gestational hypertension and preeclampsia are generally diagnosed after 20 weeks of gestation, the biological processes associated with HDP, including placental dysfunction and vascular abnormalities, may begin before clinical hypertension or preeclampsia becomes apparent. Nevertheless, the temporal and causal relationships between these processes and CAKUT remain uncertain. Therefore, while our adjusted findings did not provide clear empirical support for an independent association between clinical HDP and CAKUT, they should be interpreted cautiously given very low certainty evidence. Future prospective studies using harmonized definitions, early-pregnancy assessments of placental and vascular function, standardized outcome ascertainment, and robust confounding control are needed to clarify these associations and their clinical relevance.

Evidence for AKI remains inconclusive. Most available studies reported crude estimates without adjustment for key maternal confounders, and heterogeneity was substantial. Crude estimates differed descriptively by diagnostic criteria, but meta-regression did not establish criteria as a significant moderator (*p* = 0.679), and substantial within-group heterogeneity persisted (KDIGO: I^2^ = 51%; AKIN: I^2^ = 83%). These findings indicate that factors beyond the diagnostic criteria continued to drive inconsistencies. Further sensitivity analyses restricted to high-quality studies eliminated heterogeneity in both subgroups (I^2^ = 0% for both), and the pooled estimates lost statistical significance regardless of the criteria used. These discrepancies are more plausibly explained by differences in ascertainment, case-mix, and residual confounding than by a true protective effect [57]. Therefore, current literature does not provide robust evidence to support an altered risk of AKI among exposed offspring.

Other sources of bias warrant further attention when interpreting the preterm subgroup findings. Apparent inverse associations in preterm cohorts should therefore be interpreted cautiously. Restricting analyses to preterm subgroups can induce both collider-stratification bias and mediation blockade. The underlying causes of preterm birth differ markedly by HDP status. HDP-related preterm deliveries are predominantly iatrogenic and occur in the late preterm period, whereas non-HDP preterm births are often spontaneous and driven by infection or inflammation [58]. This etiological divergence enriches the reference group with infants who are inherently more vulnerable to kidney injury, potentially generating a spurious inverse association. Additionally, because preterm birth can lie on the causal pathway linking HDP to kidney outcomes, conditioning on it might remove the mediation effect, leaving only a direct effect that fails to capture the full impact. These biases collectively distort risk estimates. Differential survival and competing risks may further influence AKI ascertainment, and pregnancies complicated by HDP often receive intensified perinatal monitoring and protocolized neonatal care, which could mitigate AKI risk independent of biological mechanisms. Taken together, the low methodological quality of the available studies, the sensitivity of the findings to study quality, and the potential biases in preterm subgroups all argue against a causal interpretation. Current evidence does not support a clear independent effect of maternal HDP on offspring AKI.

Maternal HDP history may serve as an epidemiological marker rather than a validated clinical predictor of offspring kidney risk. Future longitudinal studies should link standardized maternal obstetric records with longitudinal offspring health records and prospectively document HDP subtype, timing of onset, severity, and diagnostic criteria. Offspring kidney outcomes should be defined using harmonized, age-appropriate criteria, with repeated assessments when establishing chronicity is clinically relevant. Relevant perinatal, familial, and later-life factors should also be consistently recorded. Where feasible, this research-oriented data collection could be embedded within existing maternal and pediatric care pathways. However, given the low certainty of current evidence, maternal HDP exposure does not currently justify universal kidney screening or routine risk stratification of asymptomatic offspring.

Our review has several strengths. We integrate evidence across multiple regions and kidney outcomes, including several large registry-based cohorts with extended follow-up periods. Conclusions relied primarily on the adjusted estimates and were further explored using subgroup, meta-regression, and sensitivity analyses.

Several limitations should be considered. First, all included studies were observational. Although some studies adjusted for important maternal factors, including diabetes, BMI, smoking, pre-existing or chronic hypertension, and kidney disease, the covariates and their definitions varied across studies and were not assessed consistently. Thus, residual confounding due to incomplete measurement, misclassification, or differences in covariate adjustment cannot be excluded. Genetic susceptibility, paternal characteristics, and other familial factors were largely unavailable. Offspring obesity and metabolic disorders were also generally not assessed and may represent later-life mediating pathways or markers of shared familial susceptibility. Therefore, the observed association should be interpreted cautiously and should not be considered as evidence that maternal HDP is an established independent causal determinant of offspring CKD. Future life-course studies with comprehensive maternal, offspring, paternal, and familial data are needed to distinguish direct intrauterine effects from shared genetic, familial, metabolic, and environmental influences. Second, age-specific reporting was limited, and follow-up duration varied substantially across the included studies. Most studies focused on neonatal or childhood populations or reported estimates across broad age ranges, whereas adult-specific estimates were rarely available. Consequently, the available evidence was insufficient to support reliable meta-analyses across neonatal/infancy, childhood, adolescence, and adulthood, precluding formal comparison of associations between childhood and adulthood and limiting assessment of how kidney disease risks may change across the life course. Follow-up duration may also have influenced outcome ascertainment, particularly for CKD outcomes. The inconsistent reporting and substantial variation in follow-up duration may therefore have contributed to between-study heterogeneity and limited direct comparison of the reported estimates. Accordingly, the overall pooled estimates should be interpreted as summary associations across heterogeneous life stages and follow-up periods, rather than as evidence of a consistent level of risk throughout the entire life course. Future studies should routinely report mutually exclusive, age-specific estimates for these life stages and use sufficiently long follow-up to enable formal comparisons of early- and late-onset kidney outcomes. Third, definitions and ascertainment of HDP and kidney outcomes varied, contributing to heterogeneity. Although HDP subtypes share overlapping vascular, placental, inflammatory, and fetal developmental pathways, they may have different effects on offspring kidney health. In our review, limited subtype-specific adjusted data precluded mutually exclusive analyses and may have obscured subtype-specific associations. Kidney outcomes were defined using different diagnostic criteria, ascertainment methods, and severity thresholds, which limited cross-study comparability. Future studies should report adjusted risks separately for each HDP subtype and adopt harmonized outcome definitions to enhance comparability. Fourth, for adjusted analyses, study numbers were limited. Therefore, publication bias assessment could not be performed. Fifth, pooling different effect measures (ORs, RRs, and HRs) on the basis of the rare-disease assumption represents a potential methodological limitation, particularly given the varying baseline risks and follow-up durations across studies. However, the broadly consistent direction of adjusted estimates across large cohorts suggests that the principal conclusions are unlikely to be driven solely by metric choice. Results should therefore be interpreted as evidence of associations rather than definitive causal effects.

## 5. Conclusions

Maternal HDP, especially PE, was associated with a higher risk of offspring CKD, although the overall certainty of this evidence was low. The pooled adjusted estimate did not show a statistically significant association between maternal HDP and CAKUT. Evidence for offspring AKI remained inconclusive because of substantial heterogeneity and limited adjusted data. Given the observational nature of the included studies, these findings do not establish causality. Future prospective studies using harmonised definitions and robust confounding control are needed to assess these associations and determine their clinical relevance.

## Figures and Tables

**Table 1:** Characteristics of studies examining associations between hypertensive disorders of pregnancy and offspring kidney outcomes.

Study	Country	Study Design	Sample Size (Case/Control)	Exposures	Outcomes, Measure of Effect	Crude Estimate (95%CI)	Adjusted Estimate (95%CI)	Confounders Adjusted	NOS Score
Arcinue 2015 [30]	USA	Case–control	211 (109/102)	HDP overall	AKI (AKIN criteria); RR	0.76 (0.52, 1.10)	Not reported	-	8
Askenazi 2013 [32]	USA	Prospective cohort	58	PE	AKI (AKIN criteria); RR	0.09 (0.01, 1.49)	Not reported	-	6
Askenazi 2015 [31]	USA	Prospective cohort	122	PE	AKI (KDIGO criteria); RR	0.23 (0.07, 0.69)	Not reported	-	6
Bolat 2013 [33]	Turkey	Retrospective cohort	1992	PIH	AKI (AKIN criteria); RR	2.23 (1.57, 3.16)	Not reported	-	6
Burgmaier 2024 [34]	Germany	Retrospective cohort	128	PE	AKI (KDIGO criteria); RR	0.89 (0.30, 2.66)	Not reported	-	6
Daga 2017 [35]	USA	Retrospective cohort	115	PIH/PE	AKI (AKIN criteria); RR	0.97 (0.44, 2.10)	Not reported	-	6
DeFreitas 2024 [12]	4 countries	Retrospective cohort	2162	HDP overall, PE/eclampsia	AKI (KDIGO criteria); OR	HDP: 0.65 (0.49, 0.87); Pre/eclampsia:0.70 (0.49, 0.99)	HDP: 0.87 (0.63, 1.21); Pre/eclampsia:0.98 (0.68, 1.39)	GA, multiple gestation, 5 min Apgar’s score and site of delivery	7
Elmas 2017 [36]	Turkey	Case–control	64 (13/51)	PE/eclampsia	AKI (KDIGO criteria); RR	0.27 (0.04, 1.93)	Not reported	-	9
Elmas 2018 [37]	Turkey	Case–control	105 (21/84)	PE	AKI (KDIGO criteria); RR	0.75 (0.28, 2.03)	Not reported	-	9
King 2025 [38]	USA	Retrospective cohort	203	PE	AKI (KDIGO criteria); RR	0.58 (0.36, 0.93)	Not reported	-	6
Koralkar 2011 [39]	USA	Prospective cohort	229	PE	AKI (AKIN criteria); RR	0.45 (0.21, 0.96)	Not reported	-	6
Lee 2017 [40]	China	Retrospective cohort	276	PE	AKI (KDIGO criteria); RR/OR	RR: 0.43 (0.26, 0.72)	OR: 0.37 (0.14, 0.97)	GA, cesarean section, Apgar scores, arterial blood pH, umbilicalarterial catheter insertion, high-frequency ventilation support, inotropic agent use, intraventricular hemorrhage, patent ductus arteriosus, chorioamnionitis, and prenatal antibiotic use	6
Lingasubramanian 2024 [41]	USA	Retrospective cohort	80	PIH	AKI (KDIGO criteria); RR	0.96 (0.70, 1.32)	Not reported	-	6
Moraes 2023 [43]	Brazil	Retrospective cohort	155	HDP overall	AKI (KDIGO criteria); RR	1.16 (0.79, 1.72)	Not reported	-	6
Majed 2019 [42]	USA	Retrospective cohort	206	PE	AKI (KDIGO criteria); RR	0.70 (0.48, 1.01)	Not reported	-	6
Srinivasan 2018 [44]	USA	Retrospective cohort	457	PE	AKI (AKIN criteria); RR	2.35 (1.48, 3.73)	Not reported	-	6
Tabel 2014 [45]	Turkey	Case–control	50 (6/44)	PE/eclampsia	AKI (AKIN criteria); RR	1.80 (0.26, 12.50)	Not reported	-	9
ÜSTÜN 2021 [46]	Turkey	Retrospective cohort	318	PE	AKI (KDIGO criteria); RR	0.75 (0.50, 1.13)	Not reported	-	6
Crump 2019 [9]	Sweden	Nationwide cohort	4,186,615	PE	CKD; HR	1.21 (1.08, 1.36)	1.29 (1.15, 1.45)	GA, sex, birth order, offspring congenital anomalies, maternal age, education level, BMI, smoking, other hypertensive disorders and DM	9
Fagundes 2025 [15]	Brazil	Nested case–control	119 (55/64)	PE	CKD; OR	2.02 (0.96, 4.23)	1.05(1.01, 1.66) ^a^	Offspring age, AKI, extrauterine growth restriction, and respiratory distress syndrome	7
Gjerde 2020 [25]	Norway	Nationwide cohort	2,663,010	PE	CKD; OR	1.11 (0.94, 1.31)	1.26 (1.04, 1.53)	Offspring sex, malformations, maternal disease (including GDM, kidney disease, rheumatic disease and essential hypertension diagnosed before pregnancy), maternal marital status	7
Hsu 2014 [26]	USA	Population-based case–control	20,535 ^b^ (1860/18,675)	GH	CKD; OR	1.36 (1.12, 1.64)	Not reported	-	7
Lihme 2025 [10]	Denmark	Nationwide cohort	2,288,589	PE	CKD; OR/HR	OR:1.25 (1.08, 1.44);	HR: 1.21(1.03,1.43) ^c^	Offspring age, gender, birth year, and maternal age, BMI, smoking, GDM, CKD	9
Dart 2015 [23]	Canada	Population-based nested case–control	5670 (945/4725)	HDP overall	CAKUT; OR	1.00 (0.81, 1.23)	Not reported	-	9
Fang 2023 [24]	China	Case–control	4619 (73/4546)	PE	CAKUT; OR	0.65 (0.24, 1.80)	Not reported	-	7
Macumber 2017 [27]	USA	Population-based case–control	15,996 (3093/12,903)	GH	CAKUT; OR	1.26 (1.07, 1.49)	Not reported	-	9
Nishiyama 2022 [28]	Japan	Nationwide cohort	100,239	HDP overall	CAKUT; OR	1.46 (1.01, 2.11)	1.10 (0.71,1.70)	Offspring sex, GA, birthweight, maternal age, education, family income, maternal smoking, alcohol, and maternal disease (including DM, obesity, and kidney disease)	9
Chou 2023 [11]	China	Population-based cohort	1,628,706	HDP overall	CAKUT; OR	1.06 (0.76, 1.48)	1.03 (0.74, 1.43)	Birth order, sex, maternal age, ACEI or ARB use during pregnancy and maternal diseases (including GDM, thyroid disorders, CAKUT, anemia, connective tissue disorders, epilepsy or mood disorder, obesity)	9
Zhang 2024 [29]	China	Birth cohort	10,179	GH	CAKUT; OR	1.45 (1.02, 2.05)	UPJO: 1.03 (0.82, 1.78); VUR:1.39 (0.63, 3.07); PUV:1.53(0.91, 2.57)	Offspring sex, low birthweight, preterm birth, pre-pregnancy overweight, smoking, alcohol consumption, folic acid intake, and GDM	8
Shnorhavorian 2011 [47]	USA	Population-based case–control	23,365 (4673/18,692)	GH, CH	CAKUT; OR	GH: 1.18 (1.02, 1.36)	CH: 1.08 (0.79, 1.49)	Maternal age, race, parity, DM, preexisting hypertension, renal disease	9

Abbreviations: AKI, acute kidney injury; AKIN, Acute Kidney Injury Network; CAKUT, congenital anomalies of the kidney and the urinary tract; CH, chronic hypertension (without superimposed PE); CI, confidence interval; CKD, chronic kidney disease; DM, diabetes mellitus; GA, Gestational age; GDM, gestational diabetes mellitus; GH, gestational hypertension; HDP, hypertensive disorders of pregnancy; HR, hazard ratio; KDIGO, Kidney Disease: Improving Global Outcomes; NOS, Newcastle–Ottawa Scale; OR, odds ratio; PE, preeclampsia; PIH, pregnancy-induced hypertension; PUV, posterior urethral valves; RR, risk ratio; UPJO, ureteropelvic junction obstruction; VUR, vesicoureteral reflux. ^a^ The reported 95% CI was markedly asymmetric on the log scale; the authors did not respond to our request for clarification. ^b^ The original cohort comprised 22,026 participants, including 1994 cases and 20,032 controls. During follow-up, 134 cases (6.7%) and 1357 controls (6.8%) were lost to follow-up. ^c^ The effect estimate was derived from term-born offspring assessed at >1 year of age.

**Table 2:** Exploratory subgroup analysis of the crude association between HDP and offspring kidney disease.

Outcomes	Subgroup Analysis		Number of Studies	Number of Participants	Pooled OR/RR (95%CI)	I^2^	P for Interaction Tests
CKD	Gestational age	Gestational age < 37 weeks	2	118,992	1.34 (0.71, 2.51)	59%	0.91
		Gestational age ≥ 37 weeks	1	2,169,716	1.29 (1.10, 1.51)	-	
	HDP subtype	Gestational hypertension	1	20,535	1.36 (1.12, 1.64)	-	0.26
		Preeclampsia	4	9,138,333	1.21 (1.11, 1.31)	1%	
CAKUT	HDP subtype	Gestational hypertension	3	49,540	1.23 (1.11, 1.37)	0%	0.22
		Preeclampsia	1	4619	0.65 (0.24, 1.80)	-	
AKI	Gestational age	Gestational age < 37 weeks	9	1432	0.69 (0.54, 0.88)	39%	0.16
		Gestational age ≥ 37 weeks	1	58	0.09 (0.01, 1.49)	-	
	Birthweight	Birthweight < 1500 g	10	1976	0.78 (0.55, 1.11)	77%	0.13
		Birthweight ≥ 1500 g	1	58	0.09 (0.01, 1.49)	-	
	HDP subtype	Gestational hypertension/Pregnancy-induced hypertension *	3	2187	1.31 (0.69, 2.48)	73%	0.07
		Preeclampsia	12	2216	0.66 (0.45, 0.98)	72%	

Abbreviations: AKI, acute kidney injury; CAKUT, congenital anomalies of the kidney and the urinary tract; CI, confidence interval; CKD, chronic kidney disease; HDP, hypertensive disorders of pregnancy; OR, odds ratio; RR, risk ratio. * Pregnancy-induced hypertension was used by some older studies; in the present analysis, it was classified as gestational hypertension only when the original study clearly described pregnancy-onset hypertension and excluded preeclampsia/eclampsia. Otherwise, it was retained as overall HDP.

## Data Availability

The dataset supporting the conclusions of this article is included within the article and its Supplementary Materials.

## Supplementary Materials

The following supporting information can be downloaded at: https://www.mdpi.com/article/10.3390/jcm15207816/s1, Figure S1. Conceptual causal framework for the association between maternal hypertensive disorders of pregnancy and offspring kidney outcomes. Figure S2. Flow chart diagram showing the selection of articles for systematic review and meta-analysis. Figure S3. Detailed risk-of-bias assessment using ROBINS-E for studies evaluating the association between maternal hypertensive disorders of pregnancy and chronic kidney disease in offspring. Figure S4. Detailed risk-of-bias assessment using ROBINS-E for studies evaluating the association between maternal hypertensive disorders of pregnancy and congenital anomalies of the kidney and the urinary tract in offspring. Figure S5. Detailed risk-of-bias assessment using ROBINS-E for studies evaluating the association between maternal hypertensive disorders of pregnancy and acute kidney injury in offspring. Figure S6. Leave-one-out sensitivity analysis of adjusted effect estimates for the association between hypertensive disorders of pregnancy and two offspring kidney outcomes. Figure S7. Leave-one-out sensitivity analysis of crude effect estimates for the association between hypertensive disorders of pregnancy and two offspring kidney outcomes. Figure S8. Forest plot of crude risk ratios for the association between maternal hypertensive disorders of pregnancy and offspring acute kidney injury (17 studies, 4769 children). Figure S9. Leave-one-out sensitivity analysis of crude risk ratios for the association between hypertensive disorders of pregnancy and offspring acute kidney injury (17 studies). Figure S10. Funnel plot of crude effect estimates for the association between hypertensive disorders of pregnancy and offspring acute kidney injury. Supplementary Table S1. Protocol deviations and outcome modifications. Supplementary Table S2. Complete search strategies for all databases. Supplementary Table S3. Newcastle–Ottawa Scale for cohort studies. Supplementary Table S4. Newcastle–Ottawa Scale for case–control studies. Supplementary Table S5. Meta-regression of crude effect estimates for offspring acute kidney injury. Supplementary Table S6. Summary of findings. PRISMA checklist.

## Abbreviations

The following abbreviations are used in this manuscript:

aHR	Adjusted hazard ratio
AKI	Acute kidney injury
AKIN	Acute Kidney Injury Network
aOR	Adjusted odds ratio
CAKUT	Congenital anomalies of the kidney and urinary tract
CI	Confidence interval
CKD	Chronic kidney disease
GRADE	Grading of Recommendations Assessment, Development and Evaluation
HDP	Hypertensive disorders of pregnancy
HR	Hazard ratio
KDIGO	Kidney Disease: Improving Global Outcomes
NOS	Newcastle–Ottawa Scale
OR	Odds ratio
PE	Preeclampsia
RR	Risk ratio
