# Supplementary material for: Kidney Outcomes in Offspring Exposed to Hypertensive Disorders of Pregnancy: A Systematic Review and Meta-Analysis
Source: J Clin Med. 2026 Oct 10;15(20):7816. doi: 10.3390/jcm15207816 (PMC13705212; doi:10.3390/jcm15207816)
Supplement: Supplementary file 1 [file jcm-15-07816-s001.zip › jcm-4541747-supplementary.pdf]

Table of contents

Content	Page
Figure S1. Conceptual causal framework for the association between maternal hypertensive disorders of pregnancy and offspring kidney outcomes.	2
Figure S2. Flow chart diagram showing the selection of articles for systematic review and meta-analysis.	3
Figure S3. Detailed risk-of-bias assessment using ROBINS-E for studies evaluating the association between maternal hypertensive disorders of pregnancy and chronic kidney disease in offspring.	4
Figure S4. Detailed risk-of-bias assessment using ROBINS-E for studies evaluating the association between maternal hypertensive disorders of pregnancy and congenital anomalies of the kidney and the urinary tract in offspring.	5
Figure S5. Detailed risk-of-bias assessment using ROBINS-E for studies evaluating the association between maternal hypertensive disorders of pregnancy and acute kidney injury in offspring.	6
Figure S6. Leave-one-out sensitivity analysis of adjusted effect estimates for the association between hypertensive disorders of pregnancy and two offspring kidney outcomes.	7
Figure S7. Leave-one-out sensitivity analysis of crude effect estimates for the association between hypertensive disorders of pregnancy and two offspring kidney outcomes.	8
Figure S8. Forest plot of crude risk ratios for the association between maternal hypertensive disorders of pregnancy and offspring acute kidney injury (17 studies, 4769 children).	9
Figure S9. Leave-one-out sensitivity analysis of crude risk ratios for the association between hypertensive disorders of pregnancy and offspring acute kidney injury (17 studies).	10
Figure S10. Funnel plot of crude effect estimates for the association between hypertensive disorders of pregnancy and offspring acute kidney injury.	11
Supplementary Table S1. Protocol deviations and outcome modifications	12
Supplementary Table S2. Complete search strategies for all databases	13-22
Supplementary Table S3. Newcastle–Ottawa Scale for cohort studies	23-24
Supplementary Table S4. Newcastle–Ottawa Scale for case-control studies	25-26
Supplementary Table S5. Meta-regression of crude effect estimates for offspring acute kidney injury	27
Supplementary Table S6. Summary of findings	28-29
PRISMA checklist	30-34

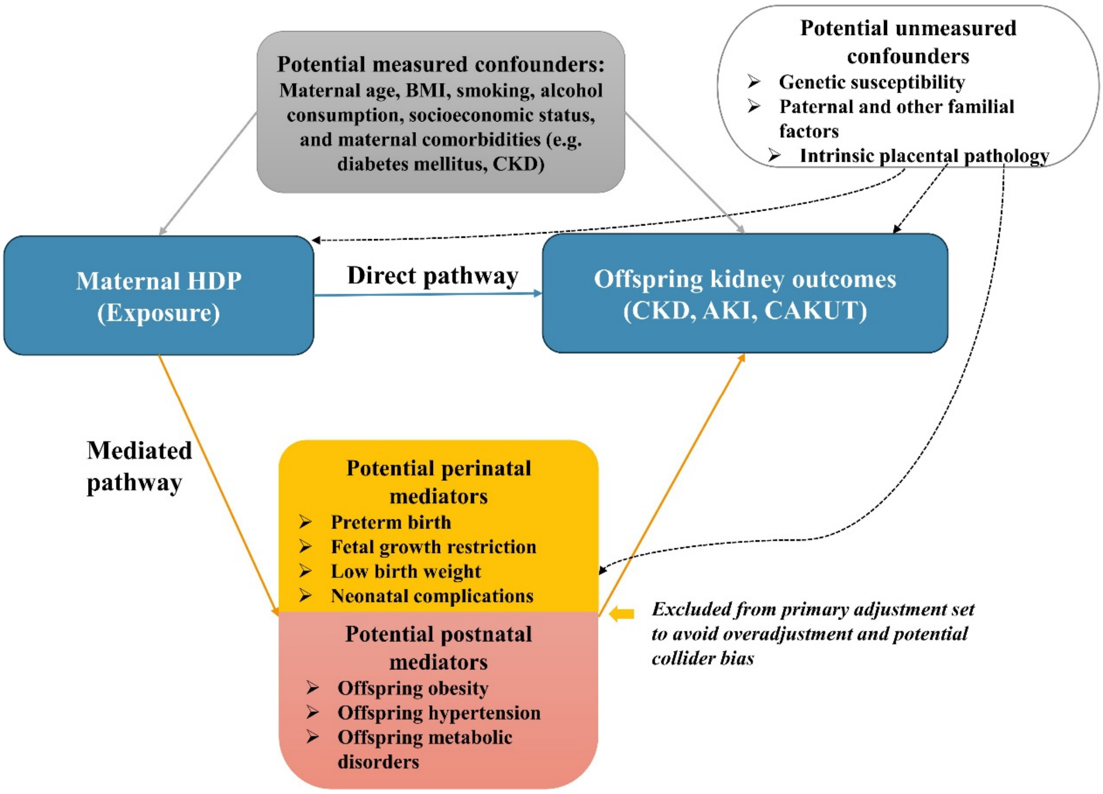

Figure S1. Conceptual causal framework for the association between maternal hypertensive disorders of pregnancy and offspring kidney outcomes. Maternal hypertensive disorders of pregnancy (HDP) were considered the exposure, and chronic kidney disease (CKD), acute kidney injury (AKI), and congenital anomalies of the kidney and urinary tract (CAKUT) in offspring were considered the outcomes. The solid blue arrow represents the hypothesized direct pathway, whereas the orange arrows represent potential indirect pathways through perinatal and postnatal mediators. Gray arrows indicate potential confounding by measured maternal characteristics, and dashed black arrows indicate possible residual or unmeasured confounding due to genetic susceptibility, paternal or other familial factors, and intrinsic placental pathology. Potential mediators were excluded from the primary adjustment set to avoid overadjustment and, under certain causal structures, potential collider bias. The diagram is a conceptual representation and does not imply that all depicted causal pathways have been empirically established. AKI, acute kidney injury; BMI, body mass index; CAKUT, congenital anomalies of the kidney and urinary tract; CKD, chronic kidney disease; HDP, hypertensive disorders of pregnancy.

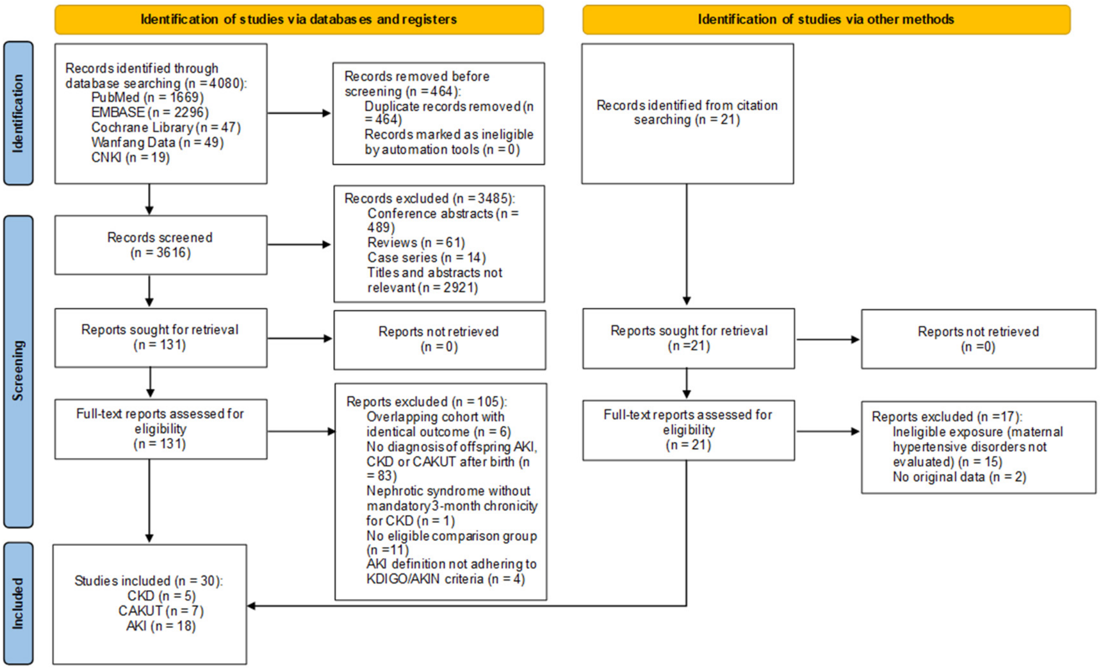

Figure S2. Flow chart diagram showing the selection of articles for systematic review and meta-analysis. AKI, acute kidney injury; AKIN, Acute Kidney Injury Network; CAKUT, congenital anomalies of the kidney and the urinary tract; CKD, chronic kidney disease; CNKI, China National Knowledge Infrastructure; KDIGO, Kidney Disease: Improving Global Outcomes.

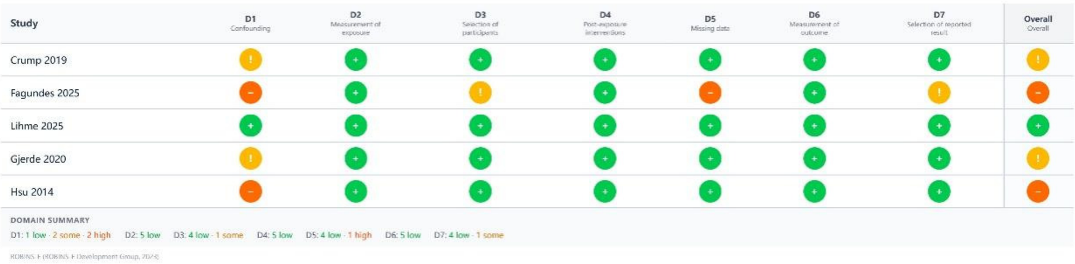

Figure S3. Detailed risk-of-bias assessment using ROBINS-E for studies evaluating the association between maternal hypertensive disorders of pregnancy and chronic kidney disease in offspring. D1–D7 represent the seven ROBINS-E domains: confounding, measurement of exposure, selection of participants, post-exposure interventions, missing data, measurement of outcome, and selection of the reported result, respectively. Green, yellow, and orange indicate low risk of bias, some concerns, and high risk of bias, respectively. “+,” “?” and “−” denote low risk of bias, some concerns, and high risk of bias, respectively. ROBINS-E, Risk Of Bias In Non-randomized Studies—of Exposure.

Study	D1 Confounding	D2 Measurement of exposure	D3 Selection of participants	D4 Post-exposure interventions	D5 Missing data	D6 Measurement of outcome	D7 Selection of reported result	Overall Overall
Nishiyama 2022	!	+	+	+	+	+	+	!
Zhang 2024	!	+	+	+	+	+	!	!
Macumber 2017	−	+	+	+	+	+	+	−
Dart 2015	−	+	+	+	+	+	!	−
Shnorhavorian 2011	+	+	+	+	+	+	+	+
Chou 2023	+	+	+	+	+	+	+	+
Fang 2023	−	+	+	+	+	+	+	−

DOMAIN SUMMARY

D1: 2 low · 2 some · 3 high D2: 7 low D3: 7 low D4: 7 low D5: 7 low D6: 7 low D7: 5 low · 2 some

ROBINS-E (ROBINS-E Development Group, 2023)

Figure S4. Detailed risk-of-bias assessment using ROBINS-E for studies evaluating the association between maternal hypertensive disorders of pregnancy and congenital anomalies of the kidney and the urinary tract in offspring. D1–D7 represent the seven ROBINS-E domains: confounding, measurement of exposure, selection of participants, post-exposure interventions, missing data, measurement of outcome, and selection of the reported result, respectively. Green, yellow, and orange indicate low risk of bias, some concerns, and high risk of bias, respectively. "+," "?" and "–" denote low risk of bias, some concerns, and high risk of bias, respectively. ROBINS-E, Risk Of Bias In Non-randomized Studies—of Exposure.

Study	D1<br>Confounding	D2<br>Measurement of exposure	D3<br>Selection of participants	D4<br>Post-exposure interventions	D5<br>Missing data	D6<br>Measurement of outcome	D7<br>Selection of reported result	Overall<br>Overall
Arcinue 2015	−	+	!	+	+	+	+	−
Askenazi 2015	−	+	!	+	+	+	+	−
Askenazi 2013	−	+	!	+	+	+	+	−
Bolat 2013	−	+	+	+	+	+	+	−
Burgmaier 2024	−	+	!	+	+	+	+	−
Daga 2017	−	+	!	+	+	+	+	−
DeFreitas 2024	−	+	+	+	+	+	+	−
Elmas 2017	−	+	!	+	+	+	+	−
Elmas 2018	−	+	!	+	+	+	!	−
King 2025	−	+	!	+	+	+	+	−
Koralkar 2011	−	+	!	+	+	+	+	−
Lee 2017	−	+	!	+	+	+	+	−
Lingasubramanian 2024	−	+	!	+	+	+	+	−
Moraes 2023	−	+	!	+	+	+	!	−
Majed 2019	−	+	!	+	+	+	+	−
Srinivasan 2018	−	+	!	+	+	+	+	−
Tabel 2014	−	+	!	+	+	+	+	−
ÜSTÜN 2021	−	+	!	+	+	+	!	−

DOMAIN SUMMARY

D1: 18 high D2: 18 low D3: 2 low · 16 some D4: 18 low D5: 18 low D6: 18 low D7: 15 low · 3 some

ROBINS-E (ROBINS-E Development Group, 2023)

Figure S5. Detailed risk-of-bias assessment using ROBINS-E for studies evaluating the association between maternal hypertensive disorders of pregnancy and acute kidney injury in offspring. D1–D7 represent the seven ROBINS-E domains: confounding, measurement of exposure, selection of participants, post-exposure interventions, missing data, measurement of outcome, and selection of the reported result, respectively. Green, yellow, and orange indicate low risk of bias, some concerns, and high risk of bias, respectively. "+," "?" and "–" denote low risk of bias, some concerns, and high risk of bias, respectively. ROBINS-E, Risk Of Bias In Non-randomized Studies—of Exposure.

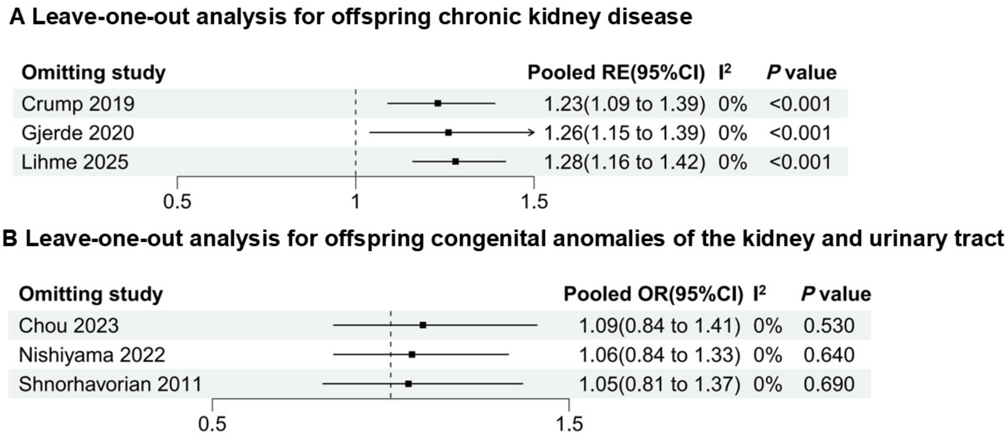

Figure S6. Leave-one-out sensitivity analysis of adjusted effect estimates for the association between hypertensive disorders of pregnancy and two offspring kidney outcomes. Because the included chronic kidney disease studies reported different effect measures, the reported ORs and HRs were synthesized in the primary analysis as a summary relative association. CI, confidence interval; OR, odds ratio; RE, relative effect.

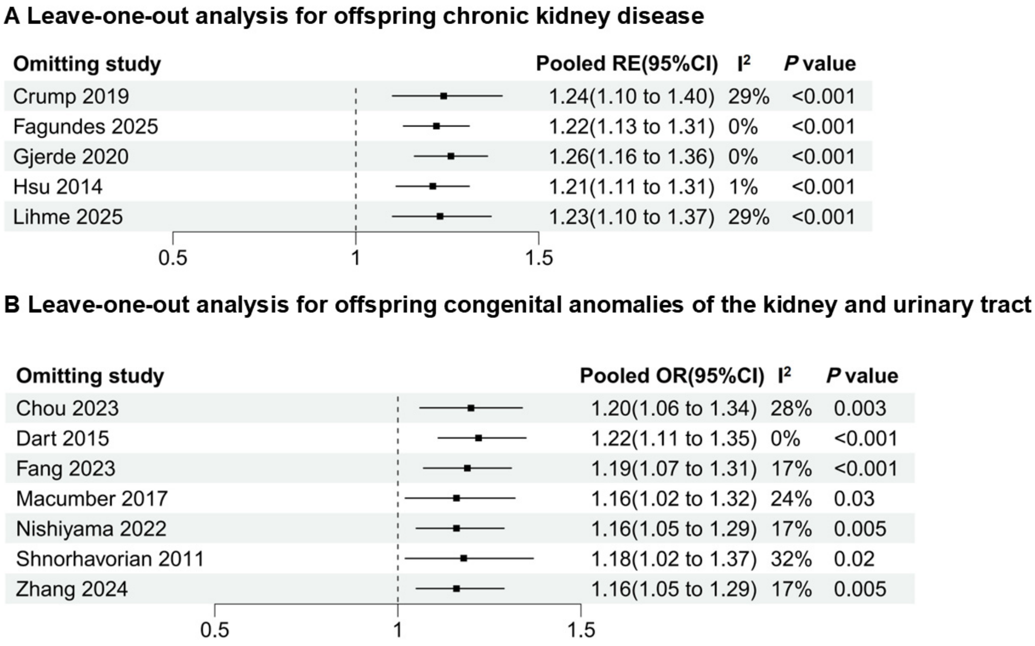

Figure S7. Leave-one-out sensitivity analysis of crude effect estimates for the association between hypertensive disorders of pregnancy and two specific offspring kidney outcomes. Because the included chronic kidney disease studies reported different effect measures, the reported odds ratios and hazard ratios were synthesized in the primary analysis as a summary relative association. CI, confidence interval; OR, odds ratio; RE, relative effect.

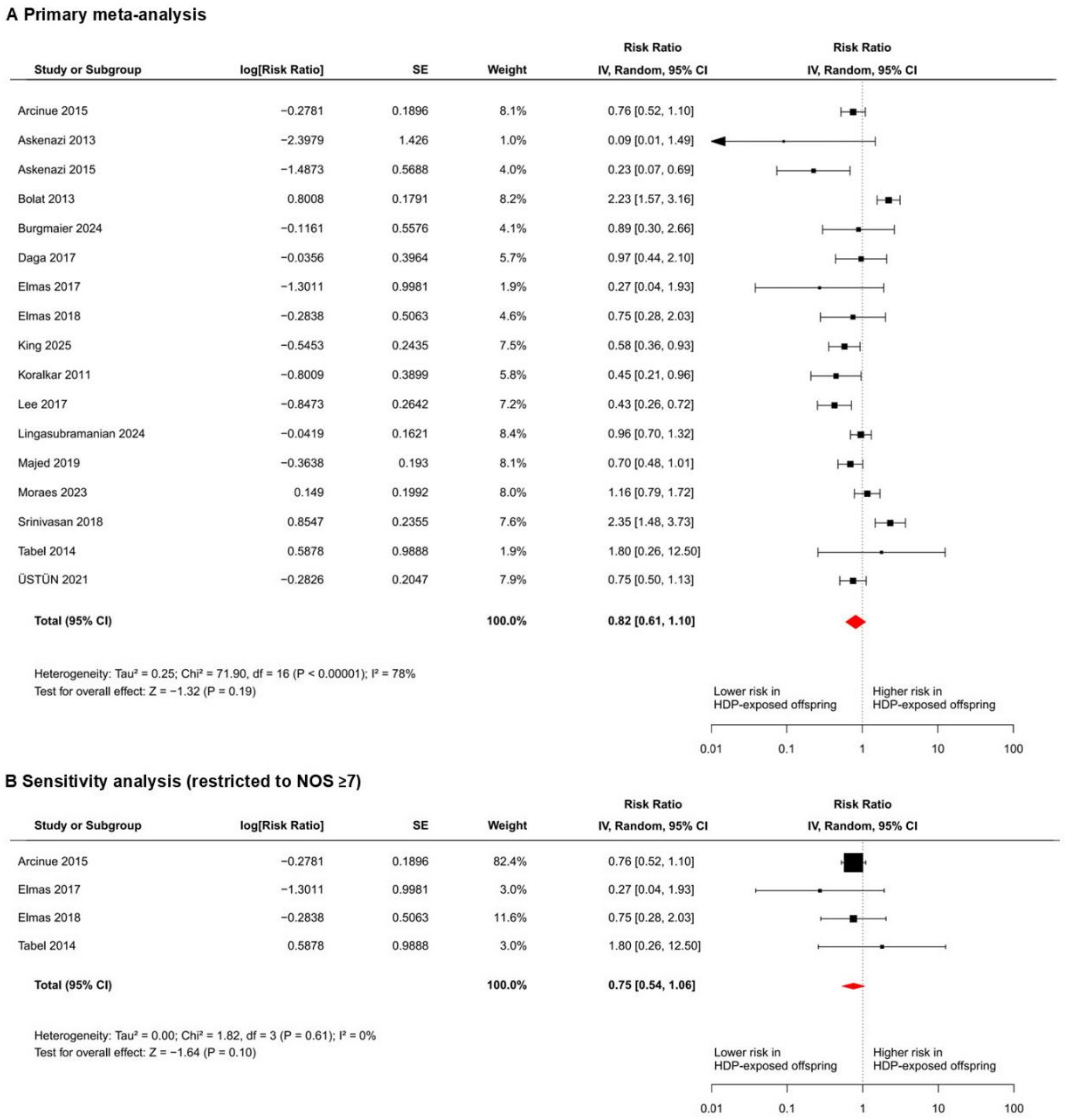

Figure S8. Forest plot of crude risk ratios for the association between maternal hypertensive disorders of pregnancy and offspring acute kidney injury (17 studies, 4769 children). Risk ratios were estimated using a random-effects model. Marker sizes reflect the weight assigned to each study (inverse variance method), and horizontal lines represent 95% confidence intervals. CI, confidence interval; IV, inverse variance; NOS, Newcastle–Ottawa Scale; SE, standard error.

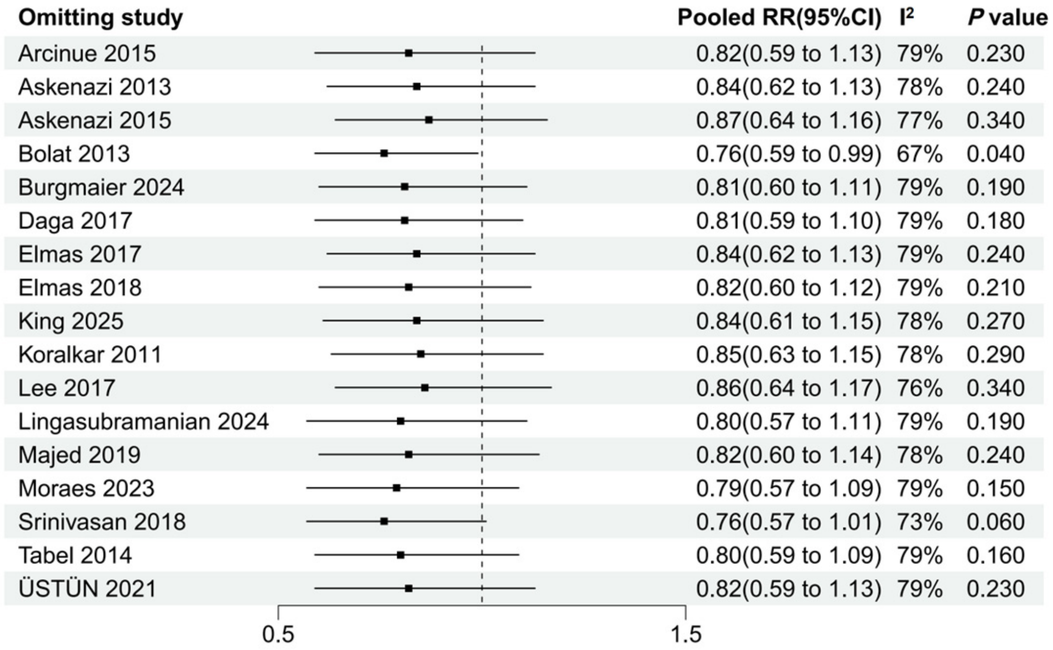

Figure S9. Leave-one-out sensitivity analysis of crude risk ratios for the association between hypertensive disorders of pregnancy and offspring acute kidney injury (17 studies). CI, confidence interval; RR, risk ratio.

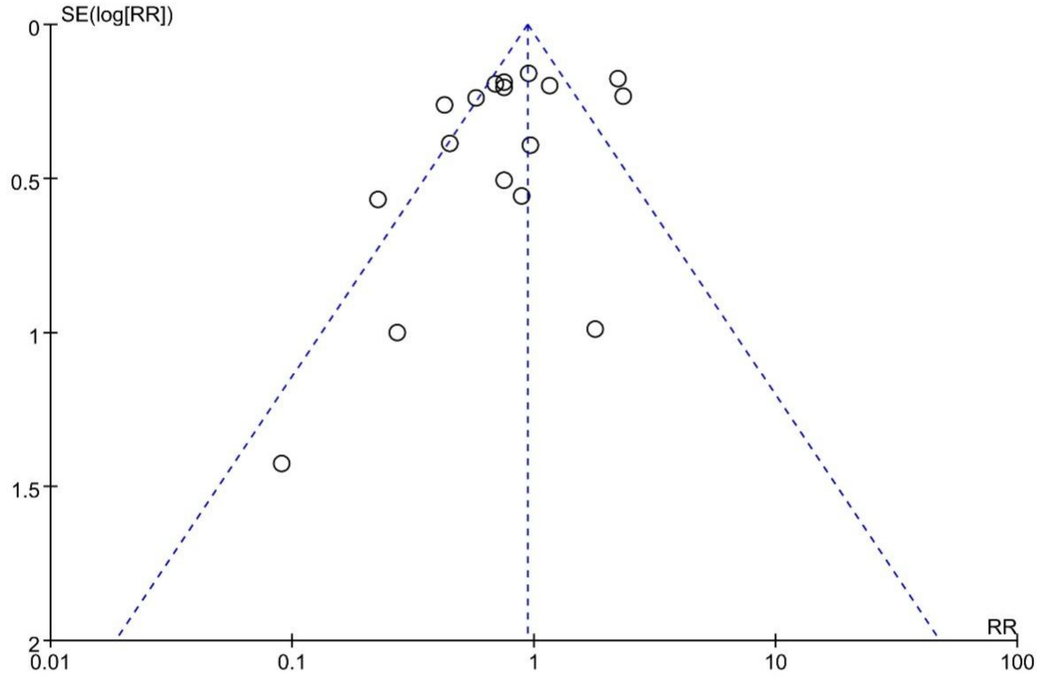

Figure S10. Funnel plot of crude effect estimates for the association between hypertensive disorders of pregnancy and offspring acute kidney injury.

Supplementary Table S1. Protocol deviations and outcome modifications

Outcome	Original status	Action	Timing	Rationale	Final status
Any kidney disease	Prespecified	Not retained as a separate outcome for quantitative and narrative synthesis	4 June 2026, during data extraction and before quantitative synthesis or access to a pooled result for this outcome	Substantial clinical and definitional heterogeneity	Removed from the final review
Glomerular and proteinuric disease	Not prespecified	Added as a post hoc exploratory outcome	4 June 2026, during data extraction and before quantitative synthesis	To explore a more specific renal phenotype	Subsequently removed
Glomerular and proteinuric disease	Post hoc exploratory outcome	Removed	7 August 2026, after receipt of the first-round peer-review comments	Potential conceptual overlap with CKD	Excluded from revised review
AKI	Prespecified	Retained	NA	NA	Primary outcome
CKD	Prespecified	Retained	NA	NA	Primary outcome
CAKUT	Prespecified	Retained	NA	NA	Primary outcome

Abbreviations: AKI, acute kidney injury; CAKUT, congenital anomalies of the kidney and the urinary tract; CKD, chronic kidney disease; NA, Not applicable

Supplementary Table S2. Complete search strategies for all databases

Database	Search number	Query
**PubMed**	#1	"Acute Kidney Injury"[Mesh]
	#2	"Acute Tubulointerstitial Nephritis" [Supplementary Concept]
	#3	Acute Renal Injuries[Title/Abstract]
	#4	Acute Renal Injury[Title/Abstract]
	#5	Acute Kidney Injuries[Title/Abstract]
	#6	Acute Renal Failure[Title/Abstract]
	#7	Renal Insufficiency, Acute[Title/Abstract]
	#8	Acute Kidney Failures[Title/Abstract]
	#9	Acute Renal Insufficiencies[Title/Abstract]
	#10	Acute Kidney Insufficiency[Title/Abstract]
	#11	Acute Renal Insufficiency[Title/Abstract]
	#12	Acute Kidney Insufficiencies[Title/Abstract]
	#13	Renal Insufficiencies, Acute[Title/Abstract]
	#14	Kidney Injury, Acute[Title/Abstract]
	#15	Acute Kidney Failure[Title/Abstract]
	#16	Kidney Insufficiency, Acute[Title/Abstract]
	#17	#1 or #2 or #3 or #4 or #5 or #6 or #7 or #8 or #9 or #10 or #11 or #12 or #13 or #14 or #15 or #16
	#18	Kidney Diseases[Mesh]
	#19	"Chronic Kidney Diseases of Uncertain Etiology"[Mesh]
	#20	CKD[Title/Abstract]
	#21	Chronic kidney disease[Title/Abstract]
	#22	Chronic Renal Failure[Title/Abstract]
	#23	Renal Insufficiency, Chronic[Mesh]
	#24	Renal Replacement Therapy[Mesh]
	#25	End-Stage Kidney Disease[Title/Abstract]
	#26	ESRD[Title/Abstract]
	#27	End-Stage Renal Disease[Title/Abstract]
	#28	Disease, End-Stage Kidney[Title/Abstract]
	#29	End-Stage Renal Failure[Title/Abstract]
	#30	"Hypertensive Nephropathy" [Supplementary Concept]
	#31	Diabetic Nephropathies[Mesh]
	#32	Diabetic Kidney Disease[Title/Abstract]
	#33	Nephropathies, Diabetic[Title/Abstract]
	#34	Nephropathy, Diabetic[Title/Abstract]
	#35	Diabetic Nephropathy[Title/Abstract]
	#36	Kidney Disease, Diabetic[Title/Abstract]
	#37	Albuminuria[Title/Abstract]
	#38	Albuminuria[Mesh]
	#39	Proteinuria[Mesh]
	#40	Proteinuria[Title/Abstract]
	#41	Nephrotic Syndrome[Mesh]
	#42	Syndrome, Nephrotic[Title/Abstract]
	#43	Nephrotic Syndromes[Title/Abstract]
	#44	Steroid Dependent Nephrotic Syndrome[Title/Abstract]
	#45	Steroid-Resistant Nephrotic Syndrome[Title/Abstract]

Database	Search number	Query
	#46	Childhood Idiopathic Nephrotic Syndrome[Title/Abstract]
	#47	Frequently Relapsing Nephrotic Syndrome[Title/Abstract]
	#48	Steroid-Sensitive Nephrotic Syndrome[Title/Abstract]
	#49	Multi-Drug Resistant Nephrotic Syndrome[Title/Abstract]
	#50	Glomerulonephritis[Mesh]
	#51	Glomerulonephritis[Title/Abstract]
	#52	"Nephritis, Interstitial"[Mesh]
	#53	chronic tubule-interstitial nephritis[Title/Abstract]
	#54	#18 or #19 or #20 or #21 or #22 or #23 or #24 or #25 or #26 or #27 or #28 or #29 or #30 or #31 or #32 or #33 or #34 or #35 or #36 or #37 or #38 or #39 or #40 or #41 or #42 or #43 or #44 or #45 or #46 or #47 or #48 or #49 or #50 or #51 or #52 or #53
	#55	Congenital Anomalies Of Kidney And Urinary Tract [Title/Abstract]
	#56	CAKUT [Title/Abstract]
	#57	#55 or #56
	#58	#17 or #54 or #57
	#59	Eclampsia[Mesh]
	#60	Hypertension, Pregnancy-Induced[Mesh]
	#61	Pre-Eclampsia[Mesh]
	#62	Pre eclampsia[Title/Abstract]
	#63	Preeclampsia[Title/Abstract]
	#64	Edema-Proteinuria-Hypertension[Title/Abstract]
	#65	Pregnancy-induced hypertension[Title/Abstract]
	#66	Gestational hypertension[Title/Abstract]
	#67	Hypertensive disorders of pregnancy[Title/Abstract]
	#68	Hypertensive disorders in pregnancy[Title/Abstract]
	#69	Chronic hypertension in pregnancy[Title/Abstract]
	#70	Hypertension in pregnancy[Title/Abstract]
	#71	#59 or #60 or #61 or #62 or #63 or #64 or #65 or #66 or #67 or #68 or #69 or #70
	#72	"Cohort Studies"[Mesh]
	#73	cohort study[Title/Abstract]
	#74	Case-Control Studies[Mesh]
	#75	Case-Control Studies[Title/Abstract]
	#76	#72 or #73 or #74 or #75
	#77	#58 AND #71 AND #76

Database	Search number	Query
EMBASE	#1	'acute kidney failure'/exp
	#2	'acute tubulointerstitial nephritis'/exp
	#3	'acute renal injuries':ti,ab
	#4	'acute kidney failure':ti,ab
	#5	'acute kidney injury':ti,ab
	#6	'acute kidney insufficiency':ti,ab
	#7	'acute renal failure':ti,ab
	#8	'acute renal insufficiency':ti,ab
	#9	'acute renal insufficiencies':ti,ab
	#10	'kidney acute failure':ti,ab
	#11	'kidney failure, acute':ti,ab
	#12	'kidney insufficiency, acute':ti,ab
	#13	'renal insufficiency, acute':ti,ab
	#14	#1 OR #2 OR #3 OR #4 OR #5 OR #6 OR #7 OR #8 OR #9 OR #10 OR #11 OR #12 OR #13
	#15	'chronic kidney failure'/exp
	#16	'chronic kidney disease':ti,ab
	#17	'chronic kidney disorder':ti,ab
	#18	'chronic kidney failure':ti,ab
	#19	'chronic kidney insufficiency':ti,ab
	#20	'chronic nephropathy':ti,ab
	#21	'chronic renal disease':ti,ab
	#22	'chronic renal failure':ti,ab
	#23	'chronic renal insufficiency':ti,ab
	#24	'kidney chronic failure':ti,ab
	#25	'kidney disease, chronic':ti,ab
	#26	'kidney failure, chronic':ti,ab
	#27	'kidney function, chronic disease':ti,ab
	#28	'renal insufficiency, chronic':ti,ab
	#29	'interstitial nephritis':ti,ab
	#30	'hypertensive nephropathy':ti,ab
	#31	'glomerulonephritis'/exp
	#32	'glomerulonephritis':ti,ab
	#33	'bright disease':ti,ab
	#34	'brights disease':ti,ab
	#35	'diffuse nephritis':ti,ab
	#36	'glomerular nephritis':ti,ab
	#37	'glomerulo nephritis':ti,ab
	#38	'kidney glomerular disease':ti,ab
	#39	'kidney glomerulus disease':ti,ab
	#40	'nephritis, diffuse':ti,ab
	#41	'nephritis, glomerular':ti,ab
	#42	'nephrotic syndrome'/exp
	#43	'nephrotic syndrome':ti,ab
	#44	'edema hypertension proteinuria syndrome':ti,ab
	#45	'oedema hypertension proteinuria syndrome':ti,ab
	#46	'epstein syndrome':ti,ab
	#47	'idiopathic nephrotic syndrome':ti,ab

Database	Search number	Query
	#48	'toxic nephrotic syndrome':ti,ab
	#49	'proteinuria'/exp
	#50	'proteinuria':ti,ab
	#51	'digestive proteinuria':ti,ab
	#52	'tubule proteinuria':ti,ab
	#53	'albuminuria'/exp
	#54	'albuminuria':ti,ab
	#55	'diabetic nephropathy'/exp
	#56	'diabetes nephropathy':ti,ab
	#57	'diabetic kidney disease':ti,ab
	#58	'diabetic nephropathies':ti,ab
	#59	'diabetic nephropathy':ti,ab
	#60	'diabetic renal disease':ti,ab
	#61	'end stage renal disease'/exp
	#62	'esrd':ti,ab
	#63	'end stage kidney disease':ti,ab
	#64	'end stage kidney failure':ti,ab
	#65	'end stage renal disease':ti,ab
	#66	'end stage renal dysfunction':ti,ab
	#67	'end stage renal failure':ti,ab
	#68	'end stage renal impairment':ti,ab
	#69	'end stage renal insufficiency':ti,ab
	#70	'end-stage kidney disease':ti,ab
	#71	'end-stage kidney failure':ti,ab
	#72	'end-stage renal disease':ti,ab
	#73	'stage 5 kidney disease':ti,ab
	#74	'stage 5 renal disease':ti,ab
	#75	'renal replacement therapy'/exp
	#76	'renal replacement therapy':ti,ab
	#77	#15 OR #16 OR #17 OR #18 OR #19 OR #20 OR #21 OR #22 OR #23 OR #24 OR #25 OR #26 OR #27 OR #28 OR #29 OR #30 OR #31 OR #32 OR #33 OR #34 OR #35 OR #36 OR #37 OR #38 OR #39 OR #40 OR #41 OR #42 OR #43 OR #44 OR #45 OR #46 OR #47 OR #48 OR #49 OR #50 OR #51 OR #52 OR #53 OR #54 OR #55 OR #56 OR #57 OR #58 OR #59 OR #60 OR #61 OR #62 OR #63 OR #64 OR #65 OR #66 OR #67 OR #68 OR #69 OR #70 OR #71 OR #72 OR #73 OR #74 OR #75 OR #76
	#78	'congenital anomalies of kidney and urinary tract'/exp
	#79	'congenital anomalies of kidney and urinary tract':ti,ab
	#80	'cakut':ti,ab
	#81	#78 OR #79 OR #80
	#82	#14 OR #77 OR #81
	#83	'preeclampsia'/exp
	#84	'eph gestoses':ti,ab
	#85	'eph gestosis':ti,ab
	#86	'eph syndrome':ti,ab
	#87	'eph toxemia':ti,ab
	#88	'hep syndrome':ti,ab
	#89	'eclamptic toxaemia':ti,ab

Database	Search number	Query
	#90	'eclamptic toxemia':ti,ab
	#91	'eclamptogenic toxaemia':ti,ab
	#92	'eclamptogenic toxemia':ti,ab
	#93	'edema-proteinuria-hypertension gestoses':ti,ab
	#94	'edema-proteinuria-hypertension gestosis':ti,ab
	#95	'gestational toxaemia':ti,ab
	#96	'gestational toxemia':ti,ab
	#97	'gestational toxicosis':ti,ab
	#98	'gestoses':ti,ab
	#99	'gestosis':ti,ab
	#100	'gestosis, eph':ti,ab
	#101	'maternal toxemia':ti,ab
	#102	'pre eclampsia':ti,ab
	#103	'pre-eclampsia':ti,ab
	#104	'pre-eclamptic':ti,ab
	#105	'pre-eclamptic toxaemia':ti,ab
	#106	'pre-eclamptic toxemia':ti,ab
	#107	'preclampsia':ti,ab
	#108	'preeclampsia':ti,ab
	#109	'preeclamptic':ti,ab
	#110	'preeclamptic toxaemia':ti,ab
	#111	'preeclamptic toxemia':ti,ab
	#112	'pregnancy toxaemia':ti,ab
	#113	'pregnancy toxaemias':ti,ab
	#114	'pregnancy toxemia':ti,ab
	#115	'pregnancy toxemias':ti,ab
	#116	'pregnancy toxicosis':ti,ab
	#117	'proteinuric hypertension of pregnancy':ti,ab
	#118	'toxaemia gravidum':ti,ab
	#119	'toxaemia, preeclamptic':ti,ab
	#120	'toxemia during pregnancy':ti,ab
	#121	'toxemia gravidum':ti,ab
	#122	'toxemia in pregnancy':ti,ab
	#123	'toxemia, preeclamptic':ti,ab
	#124	'toxemic pregnancy':ti,ab
	#125	'toxicosis gravidarum':ti,ab
	#126	'eclampsia'/exp
	#127	'eclampsia':ti,ab
	#128	'eclampsias':ti,ab
	#129	'eclamptic':ti,ab
	#130	'eclamptic convulsion':ti,ab
	#131	'eclamptic convulsions':ti,ab
	#132	'eclamptic fits':ti,ab
	#133	'eclamptic seizure':ti,ab
	#134	'eclamptic seizures':ti,ab
	#135	'maternal hypertension'/exp
	#136	'pregnancy induced hypertension':ti,ab
	#137	'gestational hypertension':ti,ab
	#138	'hypertension during pregnancy':ti,ab

Database	Search number	Query
	#139	'hypertension in pregnancy':ti,ab
	#140	'hypertension induced by pregnancy':ti,ab
	#141	'hypertension, maternal':ti,ab
	#142	'hypertension, pregnancy induced':ti,ab
	#143	'hypertension, pregnancy-induced':ti,ab
	#144	'hypertensive disorder of pregnancy':ti,ab
	#145	'maternal hypertension':ti,ab
	#146	'pregnancy associated hypertension':ti,ab
	#147	'pregnancy hypertension':ti,ab
	#148	'pregnancy induced hypertension':ti,ab
	#149	'pregnancy-induced hypertension':ti,ab
	#150	'chronic hypertension'/exp
	#151	'chronic hypertension in pregnancy':ti,ab
	#152	#83 OR #84 OR #85 OR #86 OR #87 OR #88 OR #89 OR #90 OR #91 OR #92 OR #93 OR #94 OR #95 OR #96 OR #97 OR #98 OR #99 OR #100 OR #101 OR #102 OR #103 OR #104 OR #105 OR #106 OR #107 OR #108 OR #109 OR #110 OR #111 OR #112 OR #113 OR #114 OR #115 OR #116 OR #117 OR #118 OR #119 OR #120 OR #121 OR #122 OR #123 OR #124 OR #125 OR #126 OR #127 OR #128 OR #129 OR #130 OR #131 OR #132 OR #133 OR #134 OR #135 OR #136 OR #137 OR #138 OR #139 OR #140 OR #141 OR #142 OR #143 OR #144 OR #145 OR #146 OR #147 OR #148 OR #149 OR #150 OR #151
	#153	'cohort analysis'/exp
	#154	'analysis, cohort':ti,ab
	#155	'cohort analysis':ti,ab
	#156	'cohort fertility':ti,ab
	#157	'cohort life cycle':ti,ab
	#158	'cohort studies':ti,ab
	#159	'cohort study':ti,ab
	#160	'fertility, cohort':ti,ab
	#161	'case control study'/exp
	#162	'case control study':ti,ab
	#163	'case-control studies':ti,ab
	#164	'case-control study':ti,ab
	#165	'control study, case':ti,ab
	#166	'matched case control':ti,ab
	#167	'matched case control studies':ti,ab
	#168	'matched case control study':ti,ab
	#169	#153 OR #154 OR #155 OR #156 OR #157 OR #158 OR #159 OR #160 OR #161 OR #162 OR #163 OR #164 OR #165 OR #166 OR #167 OR #168
	#170	#82 AND #152 AND #169

Database platform: Embase via Embase.com (https://www.embase.com).
Field definition: **:ti,ab** indicates a search restricted to the title and abstract fields.
Search syntax: **/exp** indicates explosion of an Emtree term. Boolean operators OR and AND were used to combine synonyms and concept blocks. Synonym expansion: Emtree entry terms were mapped to the corresponding preferred terms. Relevant free-text synonyms, abbreviations, and spelling variants were entered explicitly.

Database	Search number	Query
**Cochrane Library**	#1	MeSH descriptor: [Acute Kidney Injury] explode all trees
	#2	("acute kidney injury"):ti,ab,kw
	#3	("acute kidney failure"):ti,ab,kw
	#4	("acute renal failure"):ti,ab,kw
	#5	("acute renal injury"):ti,ab,kw
	#6	("acute kidney insufficiency"):ti,ab,kw
	#7	("acute renal insufficiency"):ti,ab,kw
	#8	("kidney failure, acute"):ti,ab,kw OR ("renal insufficiency, acute"):ti,ab,kw
	#9	#1 OR #2 OR #3 OR #4 OR #5 OR #6 OR #7 OR #8
	#10	MeSH descriptor: [Renal Insufficiency, Chronic] explode all trees
	#11	MeSH descriptor: [Kidney Failure, Chronic] explode all trees
	#12	("chronic kidney disease"):ti,ab,kw OR ("chronic kidney insufficiency"):ti,ab,kw
	#13	("chronic renal disease"):ti,ab,kw OR ("chronic renal insufficiency"):ti,ab,kw
	#14	("chronic renal failure"):ti,ab,kw OR ("chronic kidney failure"):ti,ab,kw OR ("renal failure"):ti,ab,kw
	#15	MeSH descriptor: [Nephritis, Interstitial] explode all trees
	#16	("interstitial nephritis"):ti,ab,kw
	#17	MeSH descriptor: [Glomerulonephritis] explode all trees
	#18	("glomerulonephritis"):ti,ab,kw
	#19	MeSH descriptor: [Nephrotic Syndrome] explode all trees
	#20	("nephrotic syndrome"):ti,ab,kw
	#21	MeSH descriptor: [Proteinuria] explode all trees
	#22	("proteinuria"):ti,ab,kw
	#23	MeSH descriptor: [Albuminuria] explode all trees
	#24	("albuminuria"):ti,ab,kw
	#25	MeSH descriptor: [Diabetic Nephropathies] explode all trees
	#26	("diabetic nephropathy"):ti,ab,kw OR ("diabetic kidney disease"):ti,ab,kw
	#27	("hypertensive nephropathy"):ti,ab,kw OR ("hypertensive renal disease"):ti,ab,kw
	#28	MeSH descriptor: [Renal Replacement Therapy] explode all trees
	#29	("renal replacement therapy"):ti,ab,kw
	#30	("end stage renal disease"):ti,ab,kw OR ("end stage kidney disease"):ti,ab,kw OR ("end-stage renal disease"):ti,ab,kw
	#31	("ESRD"):ti,ab,kw OR ("ESKD"):ti,ab,kw
	#32	#10 OR #11 OR #12 OR #13 OR #14 OR #15 OR #16 OR #17 OR #18 OR #19 OR #20 OR #21 OR #22 OR #23 OR #24 OR #25 OR #26 OR #27 OR #28 OR #29 OR #30 OR #31
	#33	("congenital anomalies of kidney and urinary tract"):ti,ab,kw
	#34	("CAKUT"):ti,ab,kw
	#35	#33 OR #34
	#36	#9 OR #32 OR #35
	#37	MeSH descriptor: [Pre-Eclampsia] explode all trees
	#38	MeSH descriptor: [Eclampsia] explode all trees

Database	Search number	Query
	#39	MeSH descriptor: [Hypertension, Pregnancy-Induced] explode all trees
	#40	("preeclampsia"):ti,ab,kw OR ("pre-eclampsia"):ti,ab,kw OR ("preeclamptic"):ti,ab,kw OR ("pre-eclamptic"):ti,ab,kw
	#41	("eclampsia"):ti,ab,kw OR ("eclamptic"):ti,ab,kw
	#42	("gestational hypertension"):ti,ab,kw OR ("pregnancy-induced hypertension"):ti,ab,kw OR ("pregnancy induced hypertension"):ti,ab,kw
	#43	("hypertension in pregnancy"):ti,ab,kw OR ("maternal hypertension"):ti,ab,kw OR ("hypertensive disorder of pregnancy"):ti,ab,kw
	#44	("chronic hypertension in pregnancy"):ti,ab,kw
	#45	("Edema-Proteinuria-Hypertension"):ti,ab,kw
	#46	#37 OR #38 OR #39 OR #40 OR #41 OR #42 OR #43 OR #44 OR #45
	#47	MeSH descriptor: [Cohort Studies] explode all trees
	#48	("cohort study"):ti,ab,kw OR ("cohort studies"):ti,ab,kw OR ("cohort analysis"):ti,ab,kw
	#49	MeSH descriptor: [Case-Control Studies] explode all trees
	#50	("case-control study"):ti,ab,kw OR ("case-control studies"):ti,ab,kw OR ("case control study"):ti,ab,kw OR ("matched case control"):ti,ab,kw
	#51	#47 OR #48 OR #49 OR #50
	#52	#36 AND #46 AND #51

Database platform: Cochrane Library via the Cochrane Search Manager (https://www.cochranelibrary.com).
Field definition: **:ti,ab,kw** indicates a search restricted to the title, abstract, and keyword fields.
Search syntax: **MeSH descriptor: [...] explode all trees** indicates that the specified MeSH term and all of its narrower terms were searched. Boolean operators OR and AND were used to combine synonyms and concept blocks. Synonym expansion: MeSH terms were exploded to capture narrower indexed concepts. Relevant free-text synonyms, abbreviations, and spelling and hyphenation variants were entered explicitly.

Database	Search number	Query
CNKI	#1	TKA = ('急性肾损伤' + '急性肾衰竭' + '急性肾功能不全' + '急性肾功能衰竭' + '慢性肾脏病' + '慢性肾功能不全' + '慢性肾功能衰竭' + '尿毒症' + '肾小球肾炎' + '肾病综合征' + '蛋白尿' + '糖尿病肾病' + '高血压肾病' + '终末期肾病' + '肾脏替代治疗' + '先天性肾脏和尿路畸形')
	#2	TKA= ('子痫前期' + '子痫' + '先兆子痫' + '妊娠期高血压' + '妊娠期高血压疾病' + '妊娠高血压综合征' + '妊娠合并慢性高血压')
	#3	TKA = ('队列研究' + '随访研究' + '病例对照研究')
	#4	#1 AND #2 AND #3

Database platform: China National Knowledge Infrastructure (CNKI, https://kns.cnki.net).
Field definition: **TKA** indicates a combined search restricted to Title, Keywords, and Abstract.
Search syntax: The plus sign (**+**) represents the Boolean operator OR.
English translations of the CNKI search terms:
#1: acute kidney injury; acute renal failure; acute renal insufficiency; acute kidney failure; chronic kidney disease; chronic renal insufficiency; chronic renal failure; uremia; glomerulonephritis; nephrotic syndrome; proteinuria; diabetic kidney disease; hypertensive kidney disease; end-stage kidney disease; kidney replacement therapy; and congenital anomalies of the kidney and urinary tract.
#2: pre-eclampsia; eclampsia; preeclampsia; gestational hypertension; hypertensive disorders of pregnancy; pregnancy-induced hypertension syndrome; and chronic hypertension complicating pregnancy.
#3: cohort study; follow-up study; and case-control study.
Synonym expansion was enabled to capture related Chinese medical nomenclature.
Filters: Limited to academic journal articles

Database	Search number	Query
Wanfang	#1	主题:("急性肾损伤" OR "急性肾衰竭" OR "急性肾功能不全" OR "急性肾功能衰竭" OR "慢性肾脏病" OR "慢性肾衰竭" OR "慢性肾功能不全" OR "肾小球肾炎"OR "尿毒症" OR "肾病综合征" OR "蛋白尿" OR "糖尿病肾病" OR "高血压肾病" OR "终末期肾病" OR "肾替代治疗" OR "肾脏替代治疗" OR "先天性肾脏和尿路畸形")
	#2	主题:("子痫前期" OR "先兆子痫" OR "子痫" OR "妊娠期高血压" OR "妊娠高血压综合征" OR "妊娠合并慢性高血压" OR "妊娠期高血压疾病"OR "HELLP 综合征" OR "妊高症" OR "慢性高血压")
	#3	主题:("队列研究" OR "病例对照研究" OR "随访研究")
	#4	#1 AND #2 AND #3

Database platform: Wanfang Data Knowledge Service Platform (https://www.wanfangdata.com.cn).

Field definition: The field tag "主题" (Subject) in Wanfang Data automatically performs a combined search across three fields: Title, Abstract, and Keywords.

English translations of the Wanfang search terms:

#1: acute kidney injury; acute renal failure; acute renal insufficiency; acute kidney failure; chronic kidney disease; chronic renal failure; chronic renal insufficiency; glomerulonephritis; uremia; nephrotic syndrome; proteinuria; diabetic kidney disease; hypertensive kidney disease; end-stage kidney disease; renal replacement therapy; kidney replacement therapy; and congenital anomalies of the kidney and urinary tract.

#2: preeclampsia; pre-eclampsia; eclampsia; gestational hypertension; pregnancy-induced hypertension syndrome; chronic hypertension complicating pregnancy; hypertensive disorders of pregnancy; HELLP syndrome; pregnancy-induced hypertension; and chronic hypertension.

#3: cohort study; case-control study; and follow-up study.

Syntax and operators: Quotation marks (" ") were used for exact phrase matching; AND and OR represent Boolean logic operators.

Filters: Limited to academic journal articles

Supplementary Table S3. Newcastle–Ottawa Scale for cohort studies

Study	Representativeness of the exposed cohort	Selection of the non-exposed cohort	Ascertainment of exposure	Demonstration that outcome of interest was not present at baseline	Comparability of cohorts based on the design or analysis*	Assessment of outcome	Was follow-up long enough for outcomes to occur	Adequacy of follow-up of cohorts	NOS quality score (Number of stars)
Crump 2019	★	★	★	★	★★	★	★	★	9
Gjerde 2020	★	★	★	★	0	★	★	★	7
Lihme 2025	★	★	★	★	★★	★	★	★	9
Chou 2023	★	★	★	★	★★	★	★	★	9
Nishiyama 2022	★	★	★	★	★★	★	★	★	9
Zhang 2024	0	★	★	★	★★	★	★	★	8
DeFreitas 2024	★	★	★	★	0	★	★	★	7
Lee 2017	0	★	★	★	0	★	★	★	6
Askenazi 2015	0	★	★	★	0	★	★	★	6
Askenazi 2013	0	★	★	★	0	★	★	★	6
Bolat 2013	0	★	★	★	0	★	★	★	6
Burgmaier 2024	0	★	★	★	0	★	★	★	6
Daga 2017	0	★	★	★	0	★	★	★	6
King 2025	0	★	★	★	0	★	★	★	6
Koralkar 2011	0	★	★	★	0	★	★	★	6

Lingasubramanian 2024	0	★	★	★	0	★	★	★	6
Moraes 2023	0	★	★	★	0	★	★	★	6
Majed 2019	0	★	★	★	0	★	★	★	6
Srinivasan 2018	0	★	★	★	0	★	★	★	6
ÜSTÜN 2021	0	★	★	★	0	★	★	★	6

*Comparability assessment rule: Predefined based on a directed acyclic graph identifying the minimally sufficient adjustment set for maternal hypertensive disorders and offspring kidney outcomes.

One star (★) was awarded if the study controlled for primary baseline confounders, specifically maternal age and diabetes mellitus.

An extra star (★) was awarded if the study additionally controlled for other relevant confounders, such as offspring sex, maternal smoking status, socioeconomic status/education level, body mass index, or maternal chronic kidney disease.

For the comparability item, a score of 0 indicates that the study did not adequately adjust for these core baseline maternal confounders in its study design (e.g., matching) or multivariate analysis.

For representativeness of the exposed cohort, a score of 0 indicates that the exposed cohort was not truly representative of the general population (e.g., single hospital or selected clinical sample).

Supplementary Table S4. Newcastle–Ottawa Scale for case-control studies

Author(year)	Is the case definition adequate?	Representativeness of the cases	Selection of Controls	Definition of Controls	Comparability*	Ascertainment of exposure	Same method for cases/controls	Non-Response rate	NOS quality score (Number of stars)
Fagundes 2025	★	★	★	★	★	★	★	0	7
Hsu 2014	★	★	★	★	0	★	★	★	7
Fang 2023	★	★	★	★	0	★	★	★	7
Macumber 2017	★	★	★	★	★★	★	★	★	9
Shnorhavorian 2011	★	★	★	★	★★	★	★	★	9
Dart 2015	★	★	★	★	★★	★	★	★	9
Arcinue 2015	★	★	★	★	★	★	★	★	8
Elmas 2017	★	★	★	★	★★	★	★	★	9
Elmas 2018	★	★	★	★	★★	★	★	★	9
Tabel 2014	★	★	★	★	★★	★	★	★	9

*Comparability assessment rule: Predefined based on a directed acyclic graph identifying the minimally sufficient adjustment set for maternal hypertensive disorders and offspring kidney outcomes.

One star (★) was awarded if the study controlled for primary baseline confounders, specifically maternal age and diabetes mellitus.

An extra star (★) was awarded if the study additionally controlled for other relevant confounders, such as offspring sex, maternal smoking status, socioeconomic status/education level, body mass index, or maternal chronic kidney disease.

For the comparability item, a score of 0 indicates that the study did not adequately adjust for these core baseline maternal confounders in its study design (e.g., matching) or multivariate analysis.

For the non-response rate in Fagundes et al. (2025), a total of 95 of 267 eligible participants were lost to follow-up, and another 53 had incomplete data, resulting in a high proportion of excluded subjects (148/267, 55.4%). Therefore, 0 star was assigned.

Supplementary Table S5. Meta-regression of crude effect estimates for offspring acute kidney injury

Variable	Number	Coefficients	95% CI	SE	*P*-value
Year	17	-0.001	(-0.02,0.02)	0.01	0.928
Diagnosis criteria	17	0.03	(-0.13,0.19)	0.08	0.679
Continent	17	-0.01	(-0.14,0.12)	0.06	0.910
Research type	17	-0.01	(-0.20,0.19)	0.09	0.949
Gestational age	15	-0.01	(-0.05,0.03)	0.02	0.553
Birth weight	7	-0.0001	(-0.001,0.001)	0.0003	0.817
HDP subtype	17	0.02	(-0.18,0.22)	0.09	0.54

Abbreviations: CI, confidence interval; HDP, hypertensive disorders of pregnancy; SE, standard error.

Supplementary Table S6. Summary of findings

Outcomes	Relative effect (95% CI)	№ of studies	Certainty of the evidence (GRADE)
Offspring CKD associated with maternal PE (adjusted relative effect*)	Relative effect 1.26 (1.16 to 1.37)	3 non-randomised studies	⊕⊕◯◯ Low [a]
Offspring CKD associated with maternal HDP (crude relative effect*)	Relative effect 1.23 (1.14 to 1.33)	5 non-randomised studies	⊕◯◯◯ Very low[b]
Offspring AKI associated with maternal HDP (crude RR)	RR 0.82 (0.61 to 1.10)	17 non-randomised studies	⊕◯◯◯ Very low[b,c,d]
Offspring AKI associated with maternal HDP (crude RR; KDIGO criteria)	RR 0.71 (0.55 to 0.91)	10 non-randomised studies	⊕◯◯◯ Very low[b,c]
Offspring AKI associated with maternal HDP (crude RR; AKIN criteria)	RR 1.09 (0.60 to 1.96)	7 non-randomised studies	⊕◯◯◯ Very low[b,c,d]
Offspring CAKUT associated with maternal HDP (adjusted OR)	OR 1.07 (0.87 to 1.31)	3 non-randomised studies	⊕◯◯◯ Very low[d,e]
Offspring CAKUT associated with maternal HDP (crude OR)	OR 1.18 (1.07 to 1.31)	7 non-randomised studies	⊕◯◯◯ Very low[b]

GRADE Working Group grades of evidence were as follows:

High: Very confident that the true effect is close to the estimate.

Moderate: Moderately confident; true effect likely close, but possibly substantially different.

Low: Limited confidence; true effect may be substantially different.

Very low: Very little confidence; true effect likely substantially different.

*Because the included studies reported different effect measures, including ORs and hazard ratios, the pooled estimate is presented as a pooled relative effect.

AKI, acute kidney injury; AKIN, Acute Kidney Injury Network; CAKUT, congenital anomalies of the kidney and urinary tract; CI, confidence interval; CKD,

chronic kidney disease; HDP, hypertensive disorders of pregnancy; KDIGO, Kidney Disease: Improving Global Outcomes; OR, odds ratio; PE, preeclampsia; RR, risk ratio.

[a] Observational evidence started at low certainty and was not further downgraded. For risk of bias, potential concerns related to residual confounding (e.g., lack of maternal age in one study) or adjustment for potential mediators (e.g., gestational age or preterm birth) were evaluated; however, these were judged not serious at the body-of-evidence level because remaining studies controlled for major confounders and sensitivity analyses yielded materially consistent estimates. No serious unexplained inconsistency, indirectness, or imprecision was identified. Differences in covariate adjustment were assessed under risk of bias rather than indirectness because the populations, exposures, comparators, and outcomes directly addressed the review question.

[b] Downgraded by one level for serious risk of bias because crude estimates did not account for important potential confounders.

[c] Downgraded by one level for serious inconsistency because of substantial unexplained statistical heterogeneity and clinically important variation in the magnitude or direction of study estimates.

[d] Downgraded by one level for serious imprecision because the 95% confidence interval included the null value and was compatible with both no important association and a potentially clinically significant increase or decrease in risk or odds.

[e] Observational evidence started at low certainty. Potential concerns regarding residual confounding or adjustment for potential mediators (e.g., birth weight) were assessed under the risk-of-bias domain and were judged not serious at the body-of-evidence level. No serious unexplained inconsistency or indirectness was identified. Differences in covariate adjustment were assessed under risk of bias rather than indirectness because the review question was directly addressed.

Publication bias: Publication bias was not downgraded. However, the limited number of contributing studies for several outcomes restricted the ability to assess publication bias reliably; therefore, publication bias could not be excluded.

PRISMA checklist

Section and Topic	Item #	Checklist item	Location where item is reported
**TITLE**			
Title	1	Identify the report as a systematic review.	Page 1
**ABSTRACT**			
Abstract	2	See the PRISMA 2020 for Abstracts checklist.	Page 1
**INTRODUCTION**			
Rationale	3	Describe the rationale for the review in the context of existing knowledge.	Page 2
Objectives	4	Provide an explicit statement of the objective(s) or question(s) the review addresses.	Page 2
**METHODS**			
Eligibility criteria	5	Specify the inclusion and exclusion criteria for the review and how studies were grouped for the syntheses.	Page 3-4
Information sources	6	Specify all databases, registers, websites, organisations, reference lists and other sources searched or consulted to identify studies. Specify the date when each source was last searched or consulted.	Page 3
Search strategy	7	Present the full search strategies for all databases, registers and websites, including any filters and limits used.	Page 3, Table S1
Selection process	8	Specify the methods used to decide whether a study met the inclusion criteria of the review, including how many reviewers screened each record and each report retrieved, whether they worked independently, and if applicable, details of automation tools used in the process.	Page 3
Data collection	9	Specify the methods used to collect data from reports, including how many reviewers collected data from each report, whether	Page 4

Section and Topic	Item #	Checklist item	Location where item is reported
process		they worked independently, any processes for obtaining or confirming data from study investigators, and if applicable, details of automation tools used in the process.	
Data items	10a	List and define all outcomes for which data were sought. Specify whether all results that were compatible with each outcome domain in each study were sought (e.g. for all measures, time points, analyses), and if not, the methods used to decide which results to collect.	Page 4
	10b	List and define all other variables for which data were sought (e.g. participant and intervention characteristics, funding sources). Describe any assumptions made about any missing or unclear information.	Page 5
Study risk of bias assessment	11	Specify the methods used to assess risk of bias in the included studies, including details of the tool(s) used, how many reviewers assessed each study and whether they worked independently, and if applicable, details of automation tools used in the process.	Page 5,
Effect measures	12	Specify for each outcome the effect measure(s) (e.g. risk ratio, mean difference) used in the synthesis or presentation of results.	Page 4-5
Synthesis methods	13a	Describe the processes used to decide which studies were eligible for each synthesis (e.g. tabulating the study intervention characteristics and comparing against the planned groups for each synthesis (item #5)).	Page 4-5
	13b	Describe any methods required to prepare the data for presentation or synthesis, such as handling of missing summary statistics, or data conversions.	Page 4-5
	13c	Describe any methods used to tabulate or visually display results of individual studies and syntheses.	Page 5-6
	13d	Describe any methods used to synthesize results and provide a rationale for the choice(s). If meta-analysis was performed, describe the model(s), method(s) to identify the presence and extent of statistical heterogeneity, and software package(s) used.	Page 5-6
	13e	Describe any methods used to explore possible causes of heterogeneity among study results (e.g. subgroup analysis, meta-regression).	Page 6

Section and Topic	Item #	Checklist item	Location where item is reported
	13f	Describe any sensitivity analyses conducted to assess robustness of the synthesized results.	Page 6
Reporting bias assessment	14	Describe any methods used to assess risk of bias due to missing results in a synthesis (arising from reporting biases).	Page 6
Certainty assessment	15	Describe any methods used to assess certainty (or confidence) in the body of evidence for an outcome.	Page 6
**RESULTS**			
Study selection	16a	Describe the results of the search and selection process, from the number of records identified in the search to the number of studies included in the review, ideally using a flow diagram.	Page 6-7, Figure S1
	16b	Cite studies that might appear to meet the inclusion criteria, but which were excluded, and explain why they were excluded.	Page 6-7, Figure S1
Study characteristics	17	Cite each included study and present its characteristics.	Page 7, Table 1
Risk of bias in studies	18	Present assessments of risk of bias for each included study.	Page 7, Figure S3-S5, Table S2-S3
Results of individual studies	19	For all outcomes, present, for each study: (a) summary statistics for each group (where appropriate) and (b) an effect estimate and its precision (e.g. confidence/credible interval), ideally using structured tables or plots.	Table 1

Section and Topic	Item #	Checklist item	Location where item is reported
Results of syntheses	20a	For each synthesis, briefly summarise the characteristics and risk of bias among contributing studies.	Page7,11-14
	20b	Present results of all statistical syntheses conducted. If meta-analysis was done, present for each the summary estimate and its precision (e.g. confidence/credible interval) and measures of statistical heterogeneity. If comparing groups, describe the direction of the effect.	Figure1,2,3,4,S8
	20c	Present results of all investigations of possible causes of heterogeneity among study results.	Page 14-15, Table 2, S8
	20d	Present results of all sensitivity analyses conducted to assess the robustness of the synthesized results.	Page11-14, Figure 1,2, S6-S7
Reporting biases	21	Present assessments of risk of bias due to missing results (arising from reporting biases) for each synthesis assessed.	Page 16, Figure S10
Certainty of evidence	22	Present assessments of certainty (or confidence) in the body of evidence for each outcome assessed.	Page 16, Table S6
**DISCUSSION**			
Discussion	23a	Provide a general interpretation of the results in the context of other evidence.	Page 16
	23b	Discuss any limitations of the evidence included in the review.	Page 18-19
	23c	Discuss any limitations of the review processes used.	Page 18-19

Section and Topic	Item #	Checklist item	Location where item is reported
	23d	Discuss implications of the results for practice, policy, and future research.	Page 18
**OTHER INFORMATION**			
Registration and protocol	24a	Provide registration information for the review, including register name and registration number, or state that the review was not registered.	Page 3
	24b	Indicate where the review protocol can be accessed, or state that a protocol was not prepared.	Page 3
	24c	Describe and explain any amendments to information provided at registration or in the protocol.	Page 3, Table S1
Support	25	Describe sources of financial or non-financial support for the review, and the role of the funders or sponsors in the review.	Page 20
Competing interests	26	Declare any competing interests of review authors.	Page 20
Availability of data, code and other materials	27	Report which of the following are publicly available and where they can be found: template data collection forms; data extracted from included studies; data used for all analyses; analytic code; any other materials used in the review.	Page 20
